# Supplementary material for: Temporal control of self-organized pattern formation without morphogen gradients in bacteria
Source: Mol Syst Biol. 2013 Oct 8;9:697. doi: 10.1038/msb.2013.55 (PMC3817405; doi:10.1038/msb.2013.55)
Supplement: Supplementary Information — Supplementary Figures S1–16, Supplementary Tables S1–2 [file msb201355-s1.pdf]

Temporal control of self-organized pattern formation without morphogen gradients in bacteria

Stephen Payne*, Bochong Li*, Yangxiaolu Cao, David Schaeffer, Marc D. Ryser, Lingchong You

*: equal contribution

Supplemental Materials

I. **Supplementary Materials and Methods**
II. **Supplementary Movie Legends**
III. **Supplementary Figures**
IV. **Supplementary Tables**
V. **References**

I. Supplementary Materials and Methods

Materials

pLysS is described in (Studier, 1991). Briefly, the plasmid contains the T7 lysozyme coding region in the antisense orientation relative to the *ptet* promoter. This allows for only a relatively small amount of T7 lysozyme to be synthesized.

Methods

For Fig. S2, two 3-ml overnight LB cultures derived from two single BL21 DE3 colonies transformed with pET15bLCFPT7 (the activation module) and with or without pLysS were prepared. Those cultures each were diluted 1:100 in 5 3-mL 2xYT (pH = 6.5) cultures supplemented with 0, 1, 10, 100, and 1000 µM IPTG, respectively, in addition to 50 µg/ml chloramphenicol and 75 µg/ml carbenicillin. The resulting cultures were incubated at 30ºC. After 8 hrs of incubation, the cultures were diluted 1:10 in PBS, and 1 µl diluted sample was applied to a 1% agarose PBS slab on a microscope slide. Cells were imaged using a Leica DM16000B fluorescence microscope with a mercury excitation lamp at 100X objective in both the phase and CFP channels. For the CFP measurements, the excitation filter was set to 436/20, and the emission filter was set to 480/40. Average CFP intensities over cell area were obtained for cells spanning three separate frames for each culture condition using a custom MATLAB algorithm described previously (Tan et al, 2009).

The experimental results displayed in Main Fig. 1 were obtained after using the following protocol (described briefly in Fig. S3A): A 3-ml LB culture derived from a single colony of MC4100Z1 containing the full gene circuit was grown for 12 hrs at 37 ºC. Molten agar was prepared by microwaving 0.07% w/v agar in 2xYT (pH=6.5) liquid medium. While this solution was cooling, the cell culture was diluted to ~0.2 Absorbance as measured by the Victor3 multi-well fluorimeter (Perkin Elmer, Waltham, MA) (600 nm absorbance filter, 0.1 sec). Using 0.2 Absorbance as a baseline, cultures were diluted another 3000-fold. Meanwhile, the molten agar was supplemented with 50 µg/ml chloramphenicol, 75 µg/ml carbenicillin, and 1000 µM IPTG. Then, the diluted culture was again diluted 500-fold into the molten agar. Eight 5-µl droplets of the cells mixed with the soft agar were then placed at the center of each of the 8 wells on the CultureWell™ multiwell chambered coverslip (Grace Bio-Labs, Bend, OR; referred to throughout the text as the multi-well device; see Fig. S3B for a schematic).

The inkjet printing in Fig. S5 was done using the following protocol: A 3-ml LB culture derived from a single colony of MG1655 containing the full gene circuit was grown for 16 hrs at 37ºC. Molten agar was prepared by microwaving 0.3% w/v agar in 2xYT (pH=6.5) liquid medium. While this solution was cooling, the cell culture was diluted to ~0.2 Absorbance as measured by

the Victor3 multi-well fluorimeter (Perkin Elmer, Waltham, MA) (600 nm absorbance filter, 0.1 sec). These cells were then diluted another 10-fold and placed into an ink cartridge of an Epson R280 inkjet printer (Nagano, Japan). Meanwhile, the molten agar was supplemented with 50 μg/ml chloramphenicol, 75 μg/ml carbenicillin, and 1000 μM IPTG. Two aliquots of 170 μl molten agar were placed into 2 1-mm deep, 15-mm diameter wells of a CultureWell™ multiwell chambered coverslip (Grace Bio-Labs; Bend, OR, USA; Item #103310). The surface was flattened by the addition of a glass coverslip to the top. Once the agar cooled sufficiently, the glass coverslip was removed. Then, a template constructed in GIMP containing 5-mm spacing between 1-pixel diameter spots was exported to the Epson CD Printer Program, and cells were printed accordingly. Finally, a glass coverslip was then applied to seal the agar in the wells and confine the cells to a thin layer on top of the rigid agar surface.

For Fig. S6, LuxR and LuxI were knocked out from pTuLys2CMR2, giving rise to plasmids pTuLys2CMR2LuxRKO and pTuLys2CMR2LuxIKO, respectively. This was done by using PCR to amplify the linearized pTuLys2CMR2 plasmid without the appropriate coding regions for LuxR and LuxI (including their respective ribosome binding sites). The primers used for this amplification step (shown in Table S2) contained overhanging SalI restriction sites. Once amplified, SalI restriction digests were implemented, and then, ligations were performed. The transformants from the ligation reaction yielded the correct constructs: pTuLys2CMR2LuxRKO and pTuLys2CMR2LuxIKO, which were each verified via sequencing. These plasmids were then each cotransformed with pET15bLCFPT7 into MC4100Z1 competent cells to yield the two final strains used in Fig. S6.

II. Supplementary Movie Legends

Movie S1: **Movie displaying spatiotemporal dynamics for the experimental base condition.** Movie corresponding to the raw data presented in Main Fig. 1. Here, green represents the phase channel; red represents the RFP channel; and blue represents the CFP channel. The time interval between frames is 15 minutes. The exposure times for CFP and RFP were 0.5 and 1.5 seconds, respectively. The full movie spans 8-67 hours after incubation.

Movie S2: **Movie displaying mCherry spatiotemporal dynamics for the experimental base condition.** Movie derived from RFP channel in Movie S1. The color scheme is as defined in Main Fig. 1.

III. Supplementary Figures

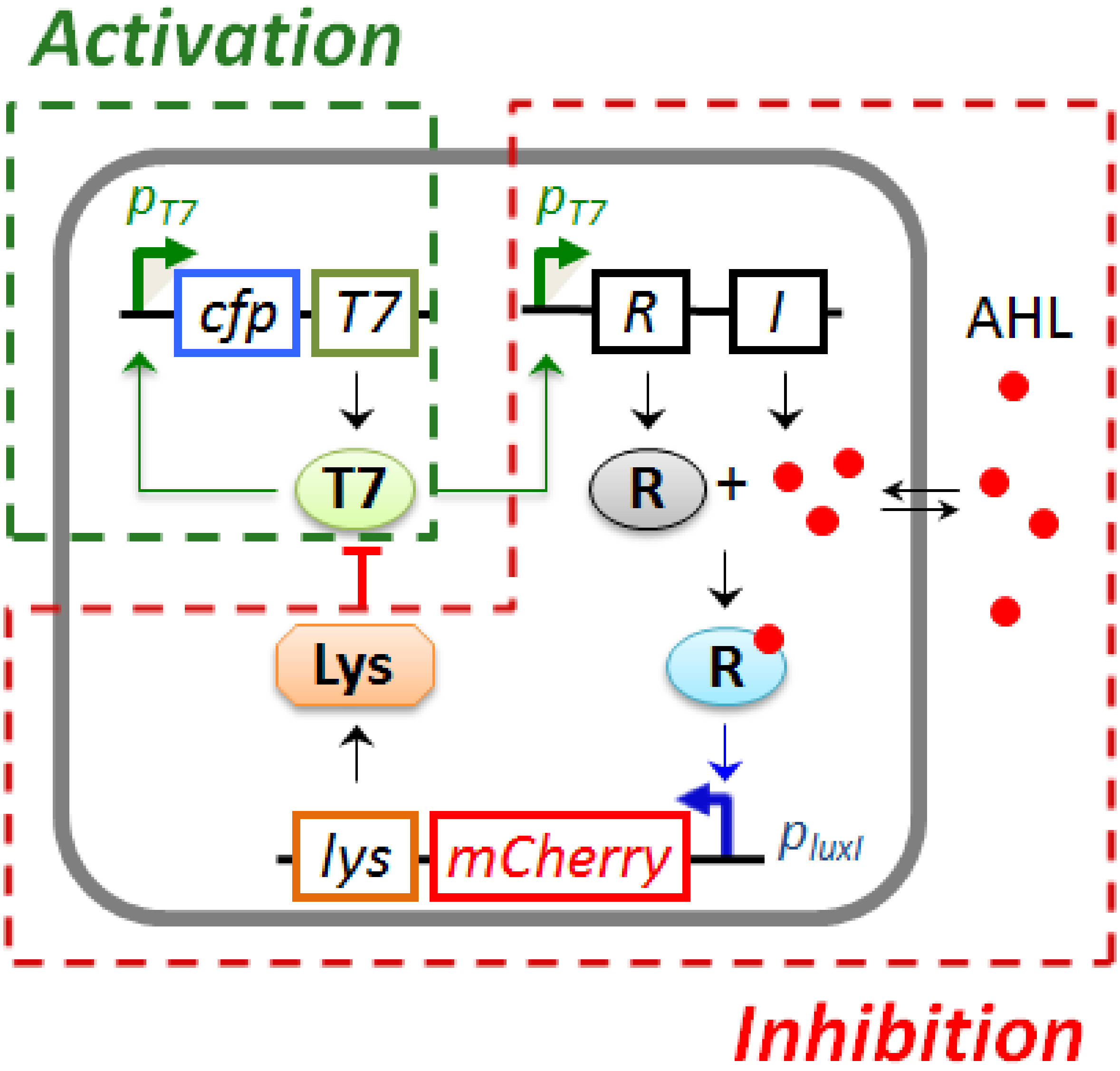

Figure S1: **Full circuit diagram.**

The activation module (green dashed box) is mediated by a mutant T7 RNA polymerase (T7), which activates itself by binding its own promoter. T7 leads to the activation of the protein LuxR (R) and a diffusible signal (AHL, red dots). AHL can diffuse freely inside and outside the cell wall. When enough intracellular AHL accumulates, AHL binds R efficiently, giving rise to a transcriptional activator complex. This complex activates expression of T7 lysozyme (Lys), which inhibits T7. Thus, the quorum-sensing mediated expression of the T7 inhibitor, Lys, constitutes the inhibition module (red dashed box). CFP and mCherry are co-expressed with T7 and Lys, respectively, as readouts of the programmed circuit dynamics.

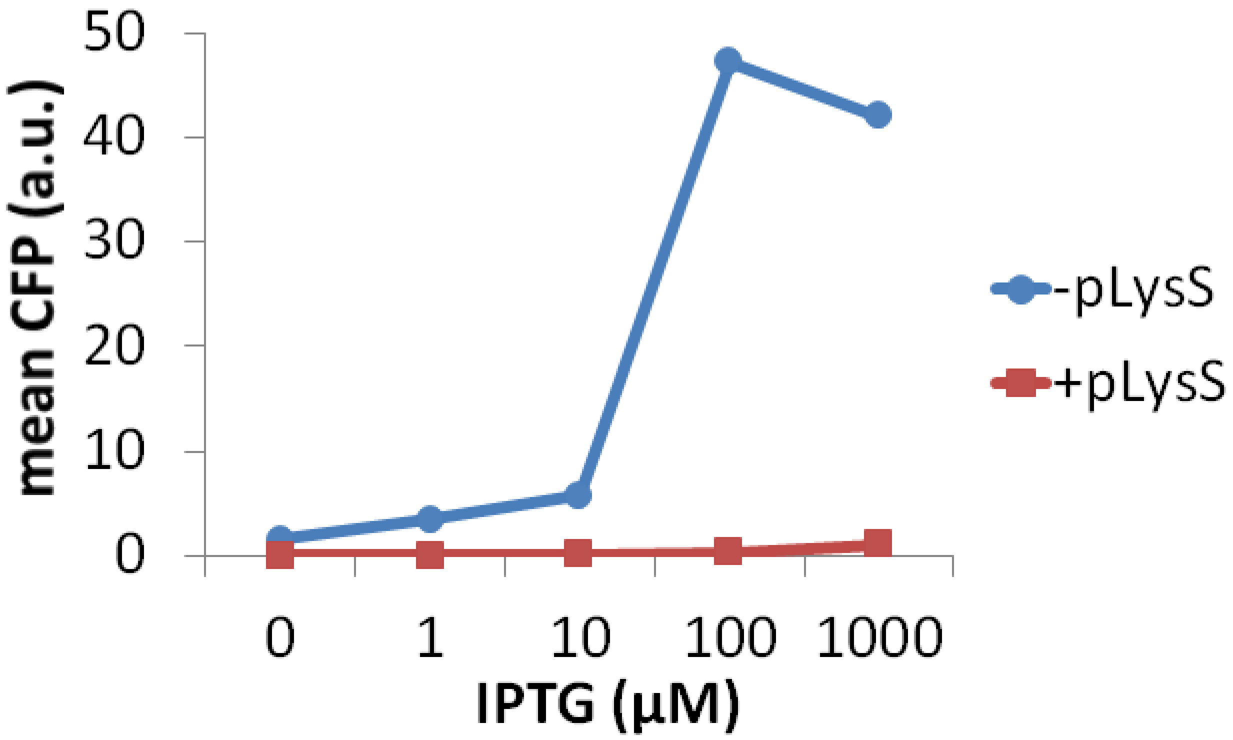

Figure S2: **T7 lysozyme inhibits the activation module.**

The mean CFP levels of the BL21 DE3 cells transformed with pET15bLCFPT7 (the activation module) are displayed for varying IPTG concentration with (red) and without (blue) pLysS after 8 hrs of growth at 30°C. For each cell included in the data analysis, background was subtracted based on the mean intensity of several hundred BL21 DE3 untransformed cells prepared in the same manner.

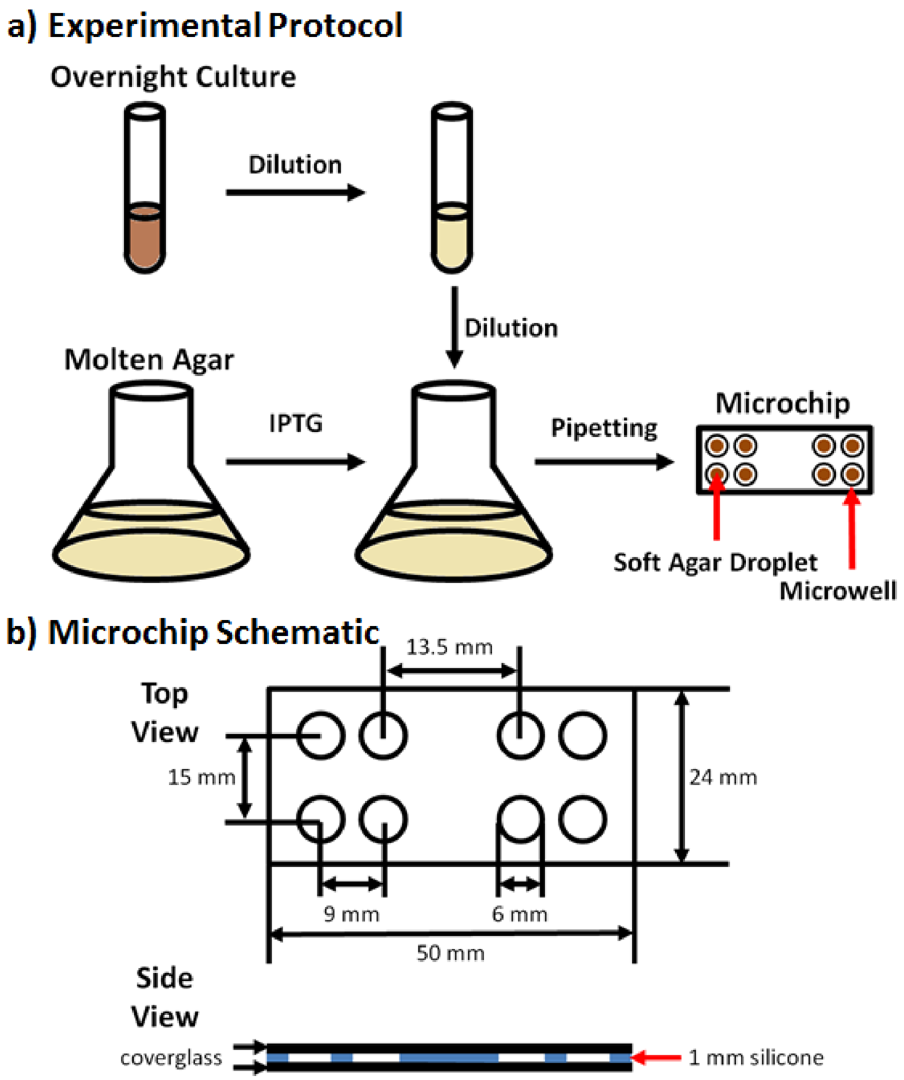

Figure S3: **Experimental setup for pattern formation demonstration.**

a) The experimental results displayed in Main Fig. 1 were obtained after using the following general protocol (see Supplementary Materials and Methods for a more thorough description): LB overnight cultures containing MC4100Z1 cells containing the full synthetic gene circuit were grown for 12 hours at 37°C. They were diluted significantly, while molten agar (0.7% in 2xYT (pH=6.5) was prepared. A small amount of diluted culture was added to the molten agar supplemented with appropriate antibiotics and IPTG once it had cooled sufficiently. Five-µl droplets from the agar were then placed into the wells of the multi-well device.

b) Schematic depicting the dimensions of the multi-well device used in the experiment. Note that another coverglass was placed on top of the multi-well device (side view) to engulf the soft agar droplets. In addition, a silicone flap in the x-direction was excised using a razor blade.

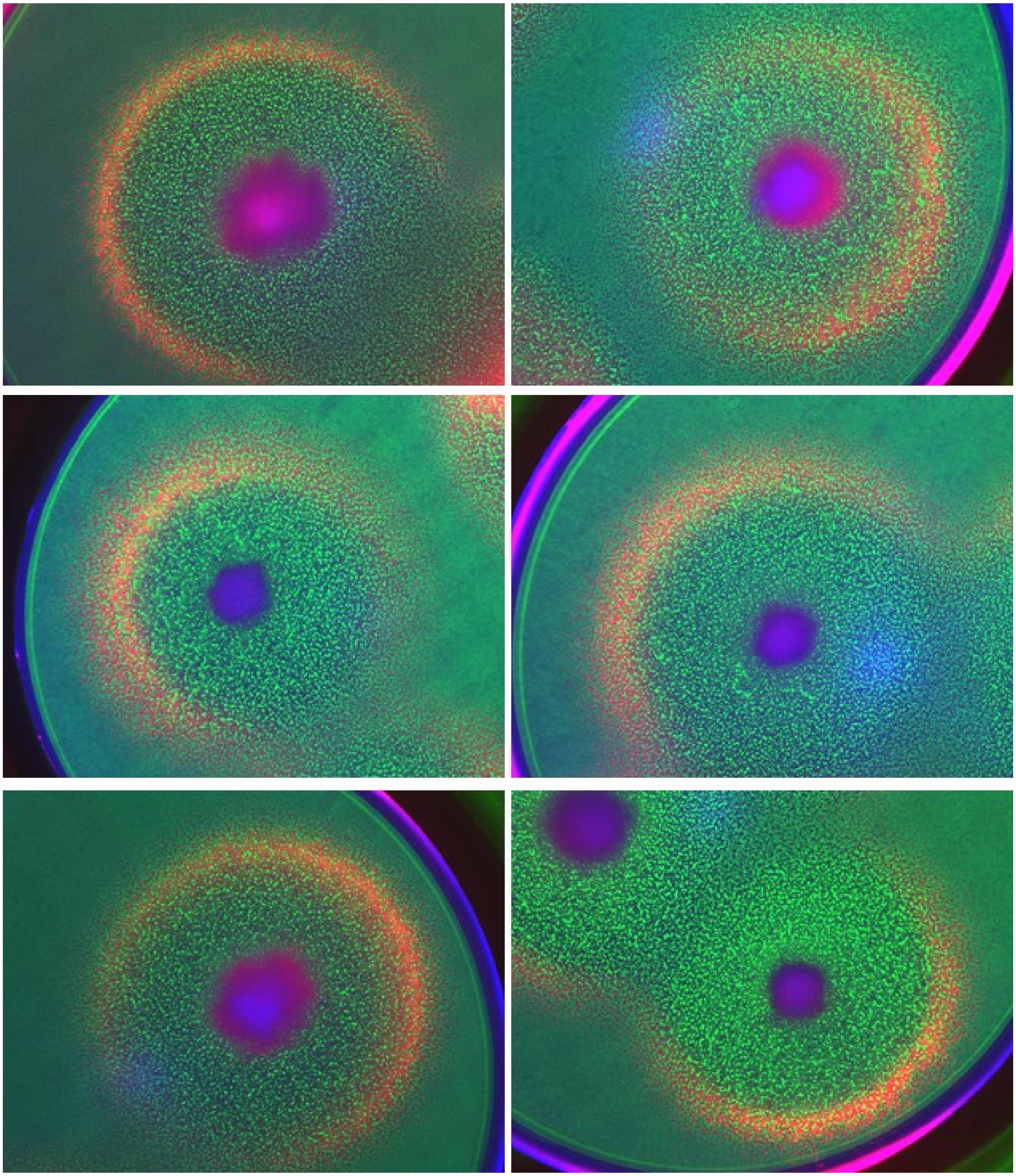

Figure S4: **Montage of patterns demonstrating reproducibility of circuit function.**

Raw 1.7 mm X 1.4 mm composite images of patterns obtained for the base-case condition described in Fig. S3 derived from six different single cell colonies, spanning five independent experiments. The color scheme is as described in Movie S1. Each pattern was imaged after 35-37 hours of incubation.

a)

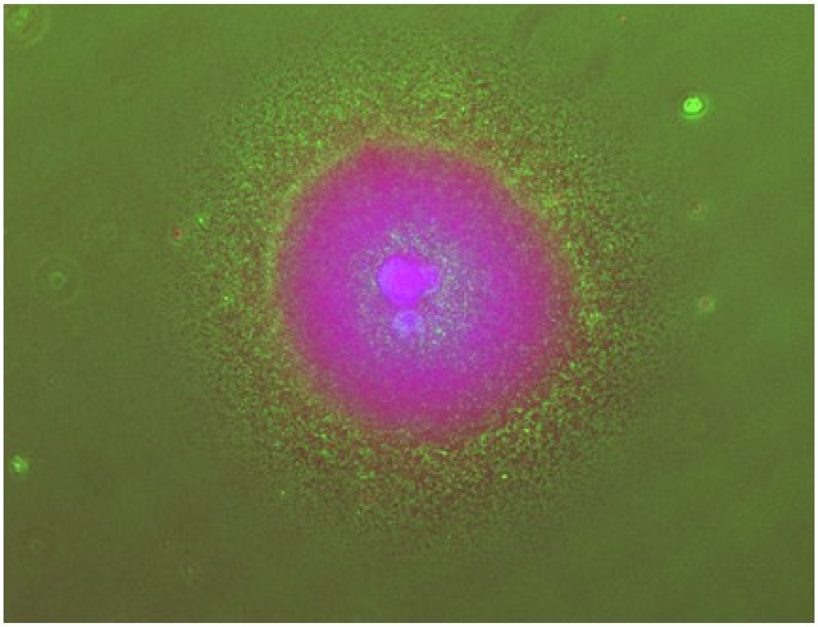

b)

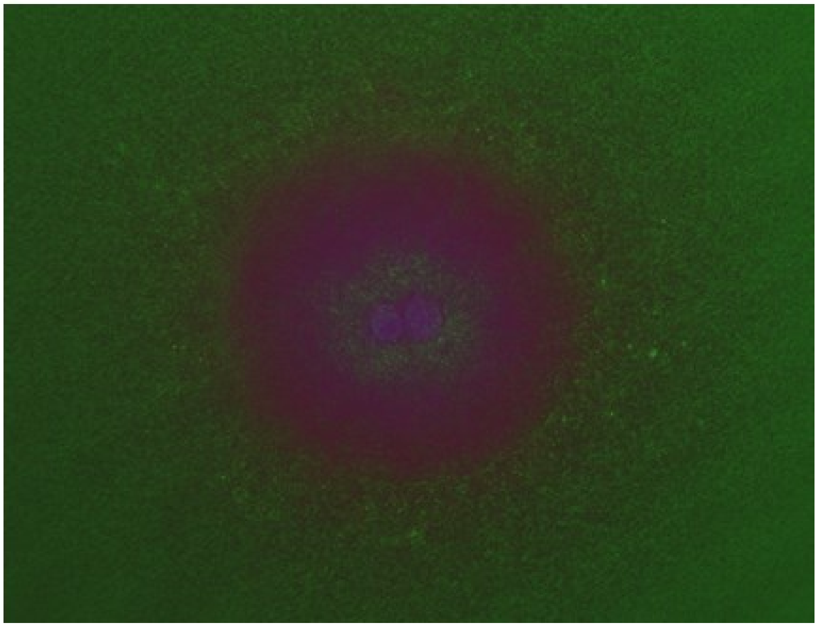

Figure S5: **Pattern formation for the synthetic gene circuit in a different cell strain under a different experimental condition.**

MG1655 (motile) cells containing the full synthetic gene circuit were printed onto a rigid surface of 2xYT (pH=6.5) medium with 0.3% agar (see Supplementary Materials and Methods for details). Raw (1.7 mm X 1.4 mm) composite images of two replicates of the printed microcolonies (a-b) were taken after 24 hours of growth. The color scheme is as described in Movie S1.

a) LuxR Knockout Replicate 1

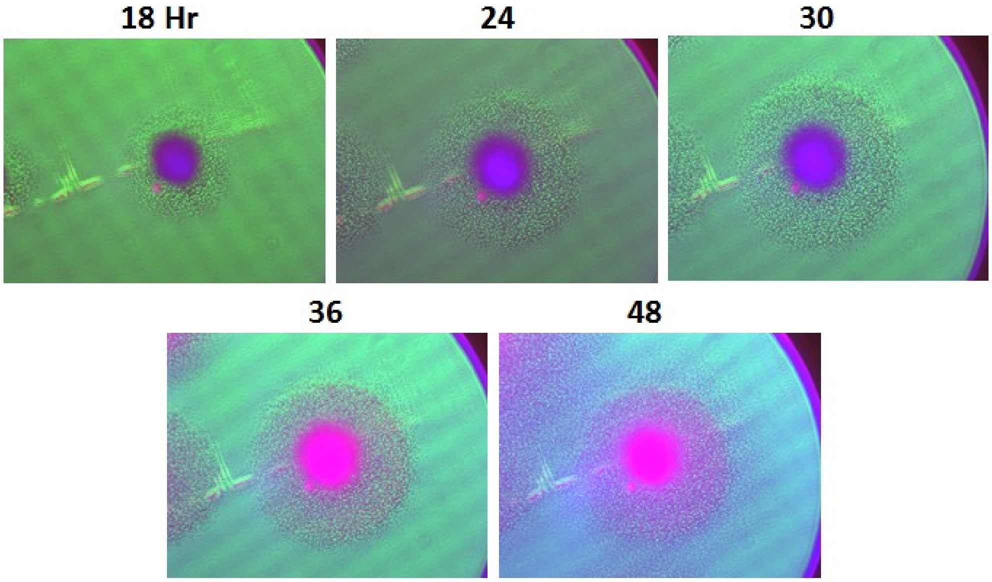

b) LuxR Knockout Replicate 2

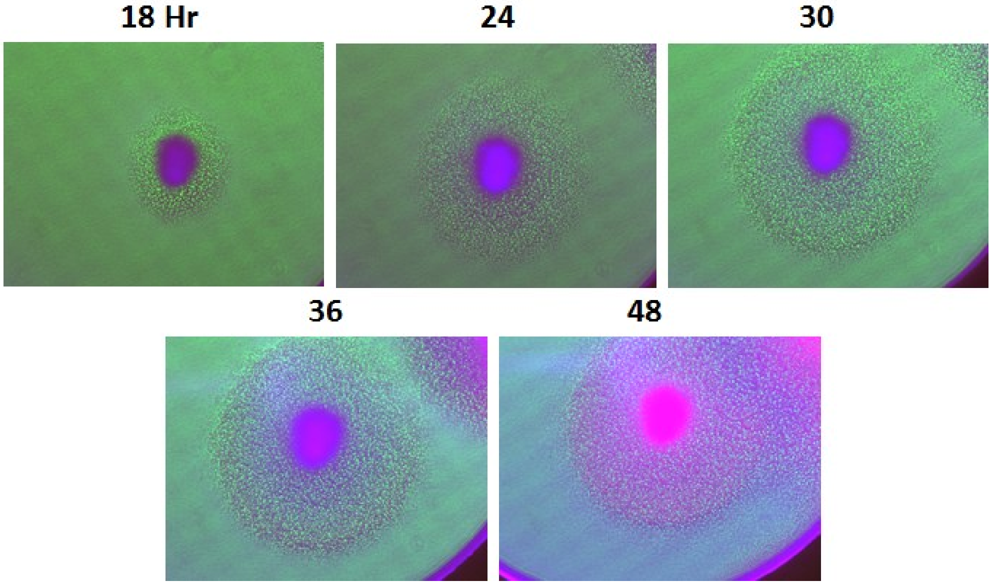

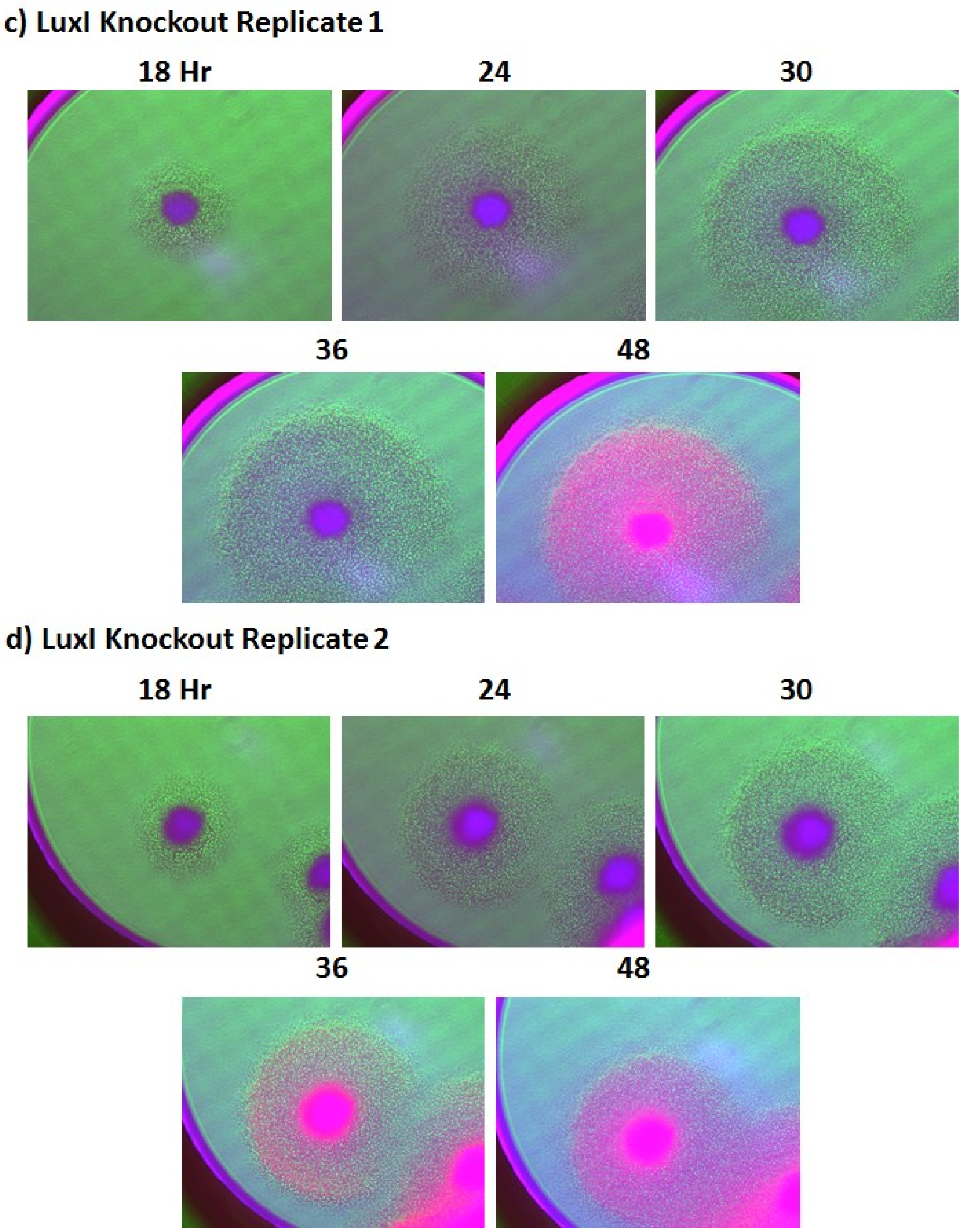

Figure S6: **Knocked out LuxR or LuxI eliminates the mCherry ring pattern.**

MC4100Z1 cells containing the full synthetic gene circuit with LuxR (a-b) or LuxI (c-b) knocked out of pTuLys2CMR2 (see Supplementary Materials and Methods for details) were placed into droplets containing 0.7% agar in 2xYT (pH=6.5) medium and grown using the same protocol implemented in Main Fig. 1. Raw (1.7 mm X 1.4 mm) composite images were obtained at 18, 24, 30, 36, and 48 hrs after incubation at 30°C for representative microcolonies. The exposure times for CFP and RFP were 0.6 and 10 seconds, respectively. The color scheme is as described in Movie S1. In each case, no discernible mCherry ring pattern is present, indicating the essentiality of AHL signaling in forming the mCherry ring.

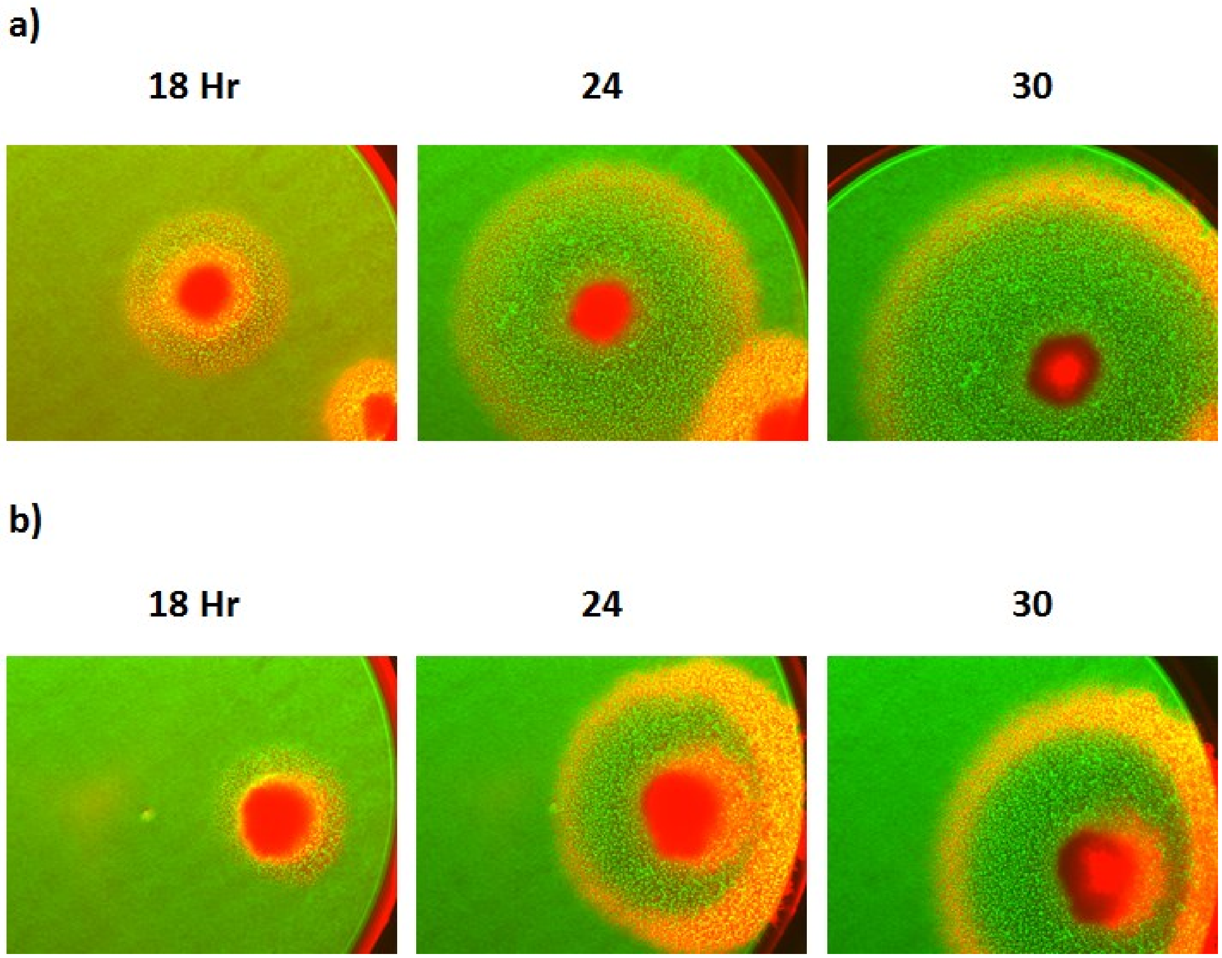

Figure S7: **Experimental demonstration of spatial-dependent gene expression.**

MC4100Z1 cells containing the plasmid ptetmCherry were placed into droplets containing 0.7% agar in 2xYT (pH=6.5) medium supplemented with 50 µg/ml chloramphenicol and 0.1 µg/ml anhydrotetracycline (aTc) and grown using the same protocol implemented in Main Fig. 1. Raw (1.7 mm X 1.4 mm) composite images were obtained at 18, 24, and 30 hrs after incubation at 30°C for two representative microcolonies (a-b). Here, green represents the phase channel, and red represents the RFP channel. Note that the mCherry ring moves with the edge of the microcolony, indicating spatial-dependent gene expression.

We note that, while the ring can form from mCherry by a single inducible promoter, this ring is fundamentally different from that formed by the full circuit. The ring in this experiment expands along with the microcolony as it reflects the spatial dependence of the gene expression capacity of the cells. In contrast, the ring formed by the full circuit is triggered by a sufficiently high concentration of AHL (the timing cue) and a sufficiently high gene expression capacity. The ring then stays at a constant size despite subsequent colony expansion, and in this manner, ring size is decoupled from later microcolony expansion (Main Figs. 1-2).

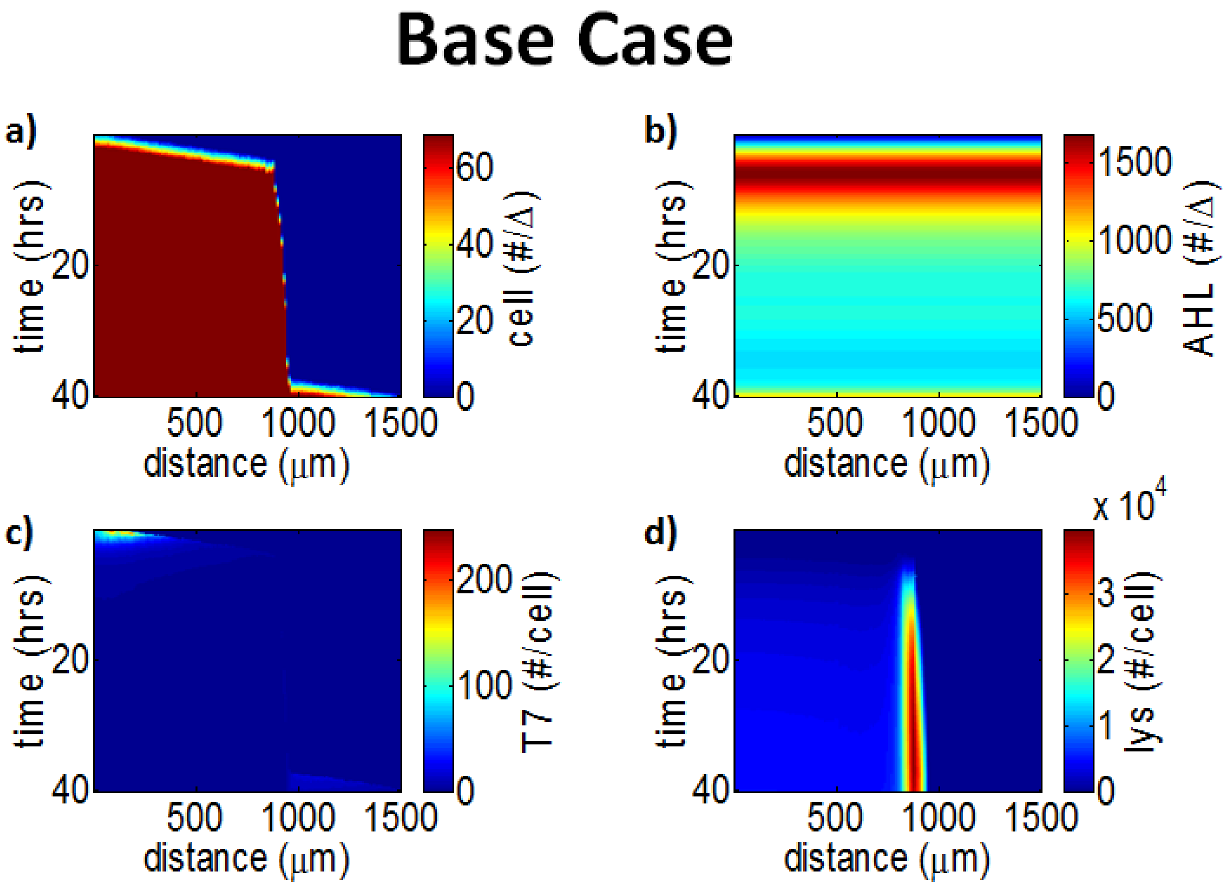

Figure S8: **Simulated spatiotemporal dynamics of key species for the base case.**

a)-d) Heat maps displaying cell density (a), AHL (b), T7 RNAP (c), and T7 lysozyme (d) for varying distance (x-axis) over time (y-axis) for the simulation shown in Main Fig. 2 (the base case). The intensity values for a)-b) represent cell and AHL numbers per spatial grid Δ, respectively, across a 1-dimensional (1D) spatial domain spanning length 300 Δ (3000 μm). The intensity values for c)-d) represent the number of T7 RNAP and T7 lysozyme molecules per cell, respectively, across the 1D spatial domain.

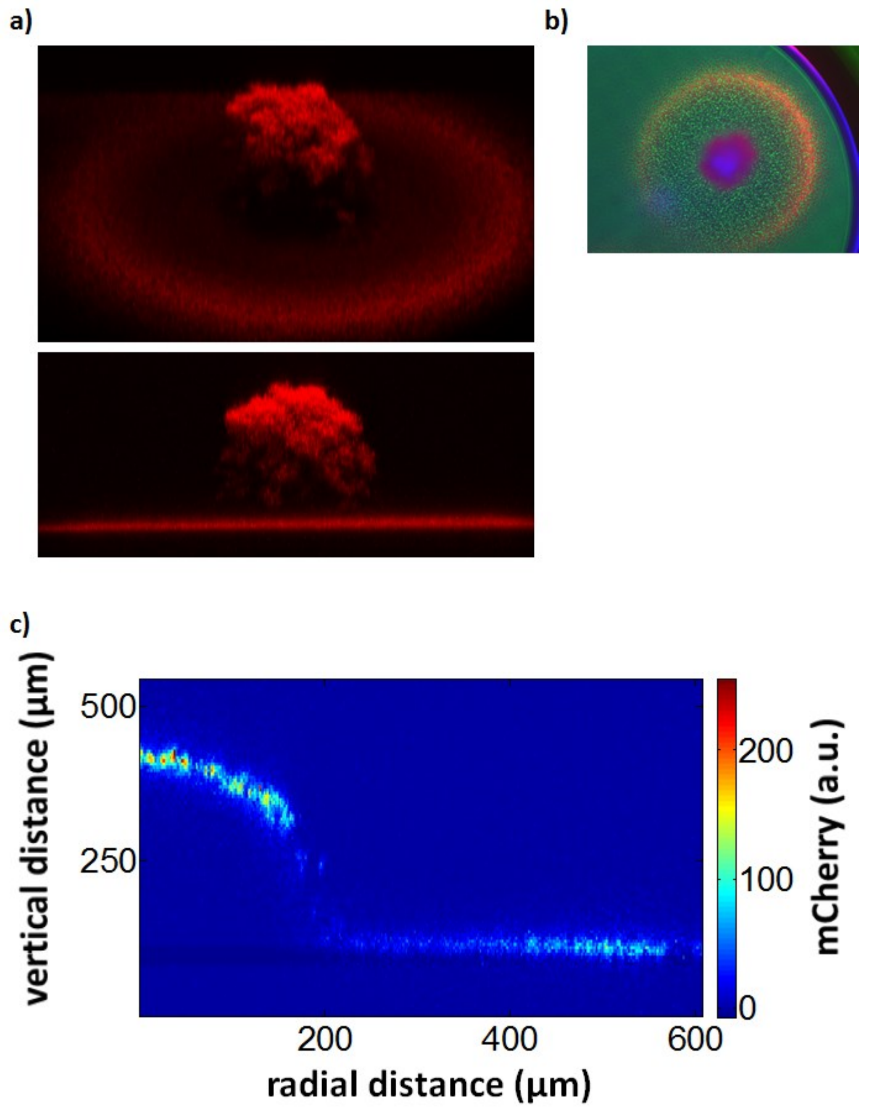

Figure S9: **3-Dimensional (3D) confocal image of a typical base-case mCherry pattern.**

a) Tilted (top) and side (bottom) views of a 3D reconstruction of the mCherry pattern based on a series of z-slice images 5.21 µm in depth spanning x- and y-dimensions of length 1214 µm taken by a Zeiss LSM 780 upright confocal microscope. The pattern was excited at a 561 nm wavelength, and the emission filter used collected wavelengths between 576 and 696 nm. The pattern was obtained for the base-case condition described in Fig. S3. The 3D reconstruction was done using MetaMorph (Molecular Devices, LLC, Sunnyvale, CA).

b) Raw 1.7 mm X 1.4 mm composite fluorescent image of the pattern displayed in a). The color scheme is as described in Movie S1.

c) Heat map displaying mCherry intensity in both the vertical (y-axis) and radial (x-axis) directions as derived from the confocal microscope image reconstructed in a). The radial intensity values are the average intensity values across angles spanning $3\pi/4$ to $7\pi/4$. Processing was carried out using a custom MATLAB code.

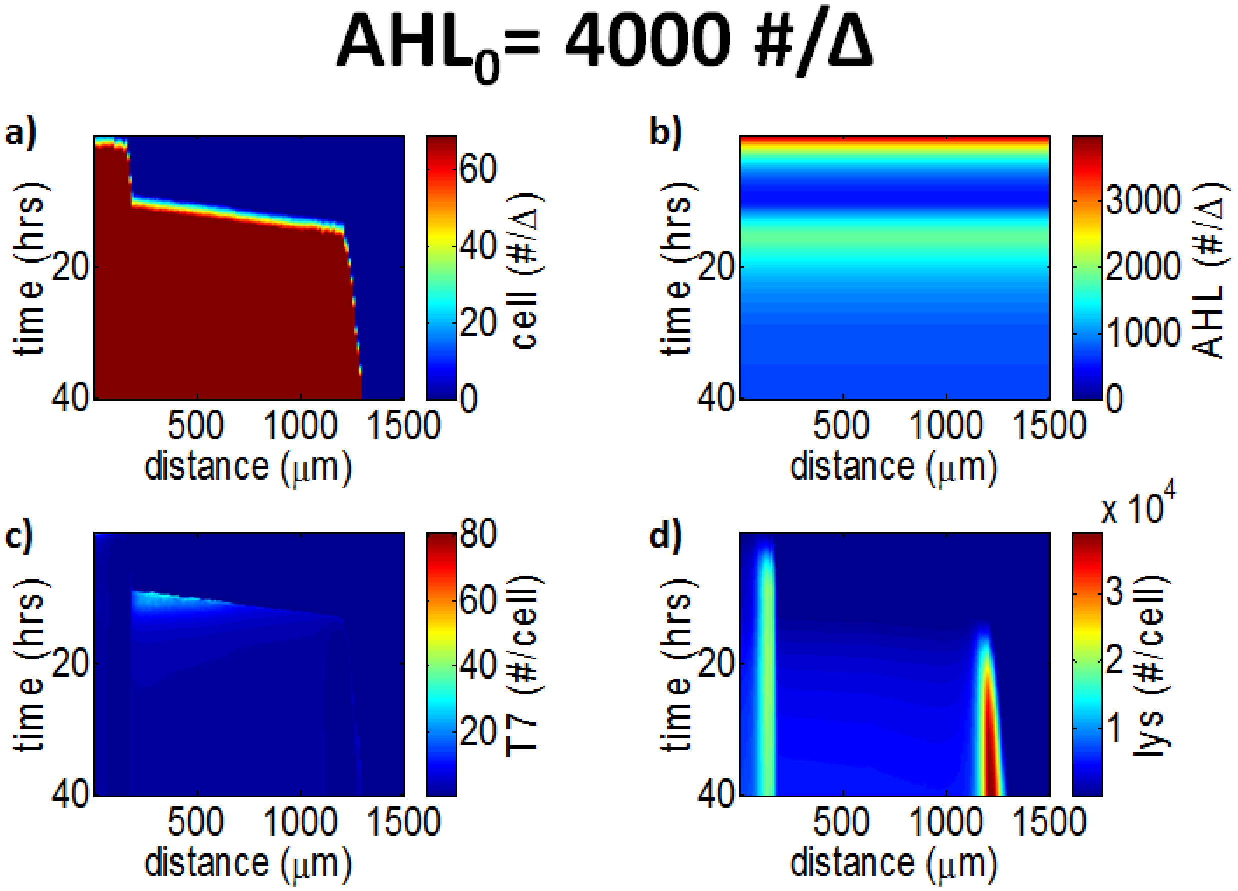

Figure S10: **Simulated spatiotemporal dynamics of key species for an initial AHL concentration of 4000 molecules/Δ.**

a)-d) Heat maps displaying cell density (a), AHL (b), T7 RNAP (c), and T7 lysozyme (d) for varying distance (x-axis) over time (y-axis) for a typical simulation with an initial AHL concentration of 4000 molecules/Δ. Units are as described in Fig. S8, and the simulation took place across a 1-dimensional (1D) spatial domain spanning length 300 Δ (3000 μm).

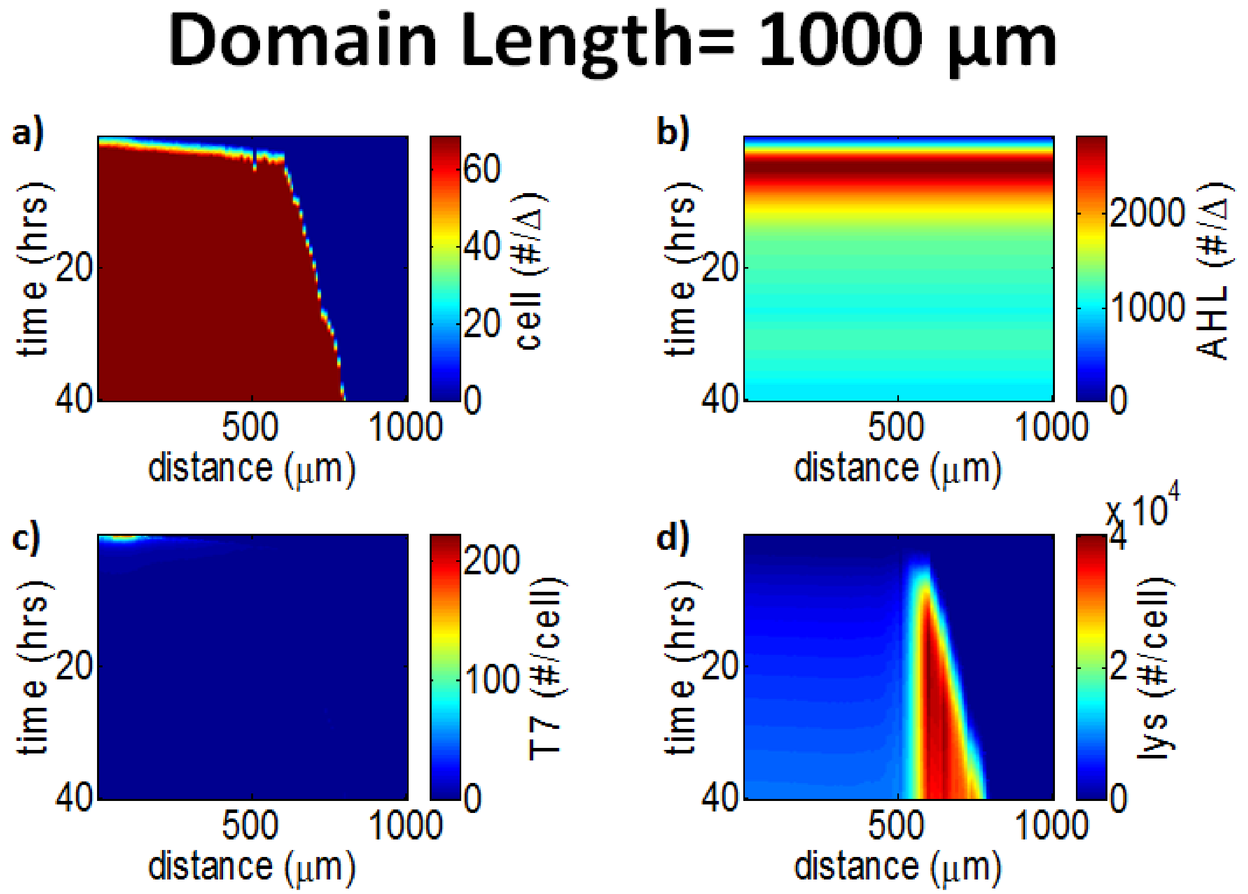

Figure S11: **Simulated spatiotemporal dynamics of key species for a domain size of 100 Δ (1000 μm).**

a)-d) Heat maps displaying cell density (a), AHL (b), T7 RNAP (c), and T7 lysozyme (d) for varying distance (x-axis) over time (y-axis) for a typical simulation with a 1-dimensional (1D) spatial domain spanning length 100 Δ (1000 μm). Units are as described in Fig. S8.

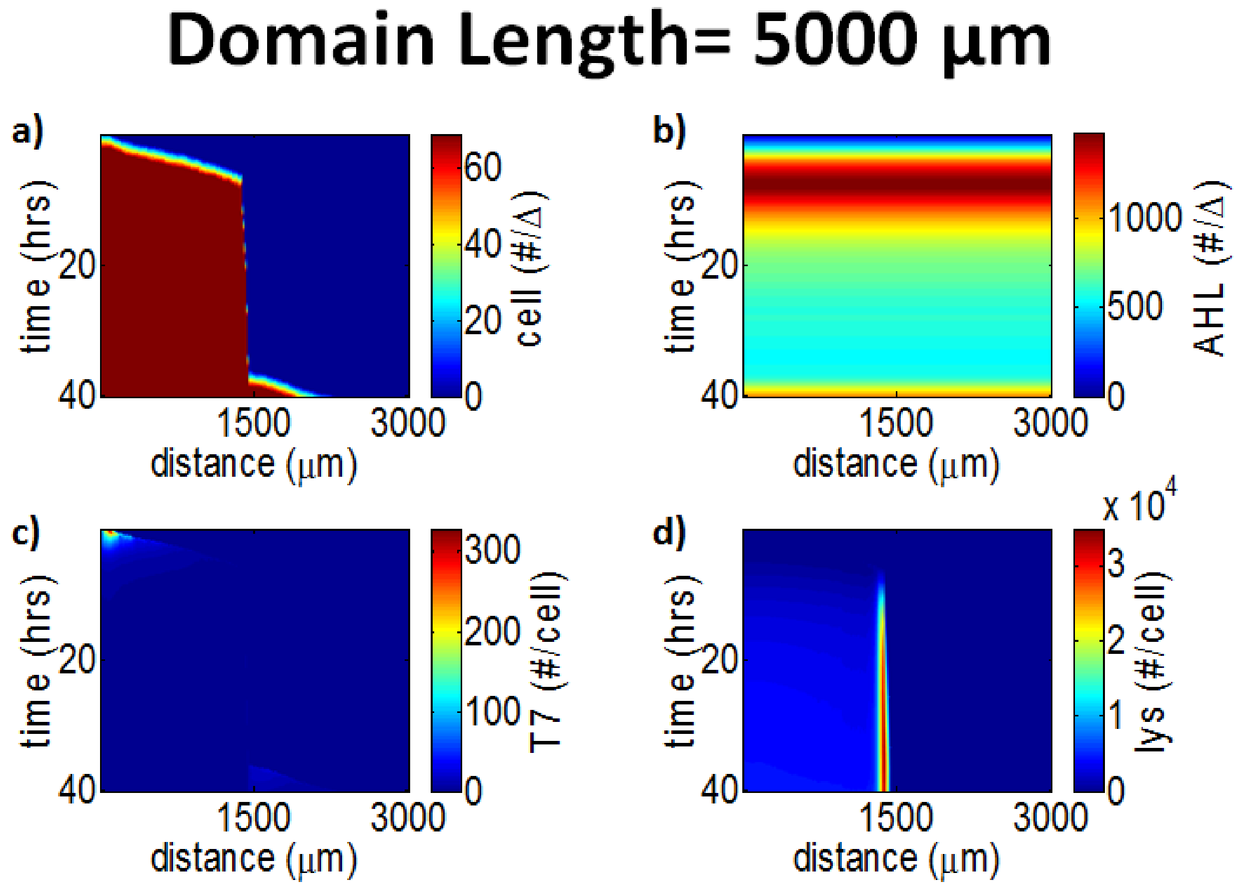

Figure S12: **Simulated spatiotemporal dynamics of key species for a domain size of 500 Δ (5000 μm).**

a)-d) Heat maps displaying cell density (a), AHL (b), T7 RNAP (c), and T7 lysozyme (d) for varying distance (x-axis) over time (y-axis) for a typical simulation with a 1-dimensional (1D) spatial domain spanning length 500 Δ (5000 μm). Units are as described in Fig. S8.

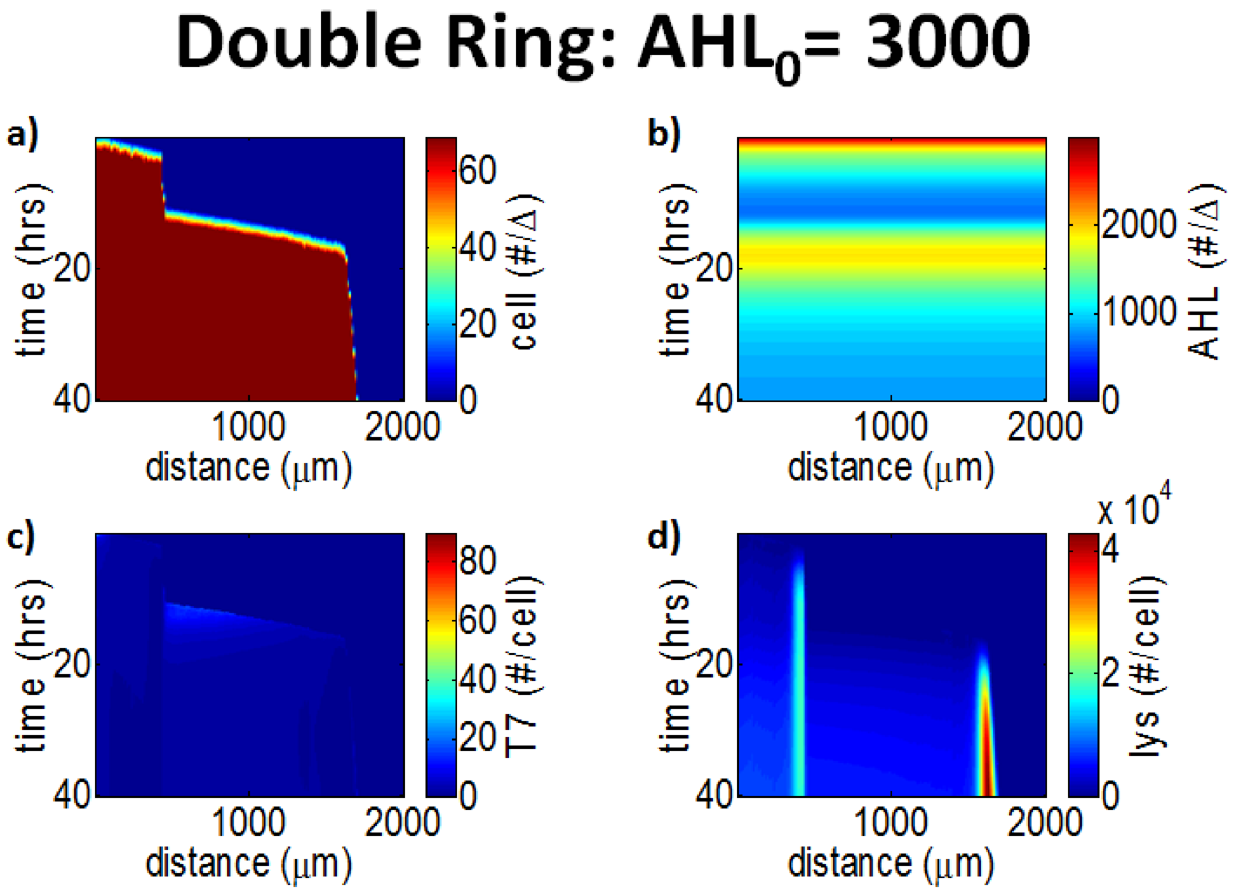

Figure S13: **Simulated spatiotemporal dynamics of key species for the double-ring case (an initial AHL concentration of 3000 molecules/Δ).**

a)-d) Heat maps displaying cell density (a), AHL (b), T7 RNAP (c), and T7 lysozyme (d) for varying distance (x-axis) over time (y-axis) for the simulation showed in Main Fig. 4 with an initial AHL concentration of 3000 molecules/Δ. Units are as described in Fig. S8, and the simulation took place across a 1-dimensional (1D) spatial domain spanning length 300 Δ (3000 μm).

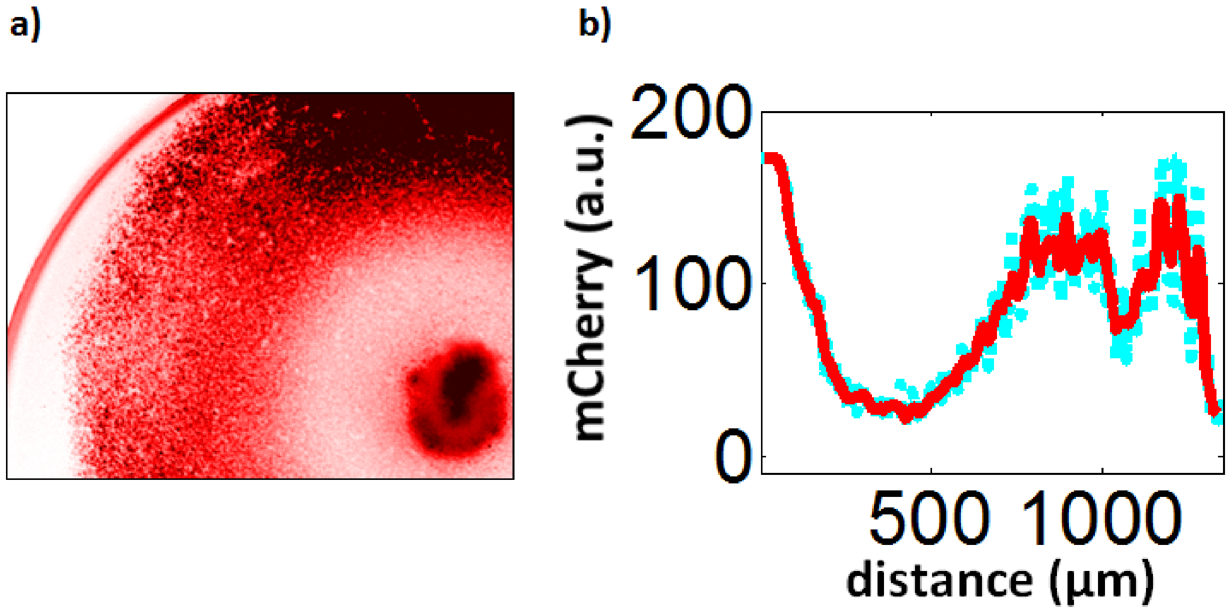

Figure S14: **An additional experimentally obtained mCherry double-ring pattern.**

a) The image was obtained after culturing a microcolony for 42 hours at an initial AHL concentration of 100 nM. The experimental protocol is as described for Main Fig. 4. The color scheme is as described in Main Fig. 1.

b) mCherry intensity (cyan dots) at varying radii for the image in a). The solid red line is the running average at varying radii. mCherry was calculated as described in Main Fig. 1 across angles spanning π to $5\pi/4$.

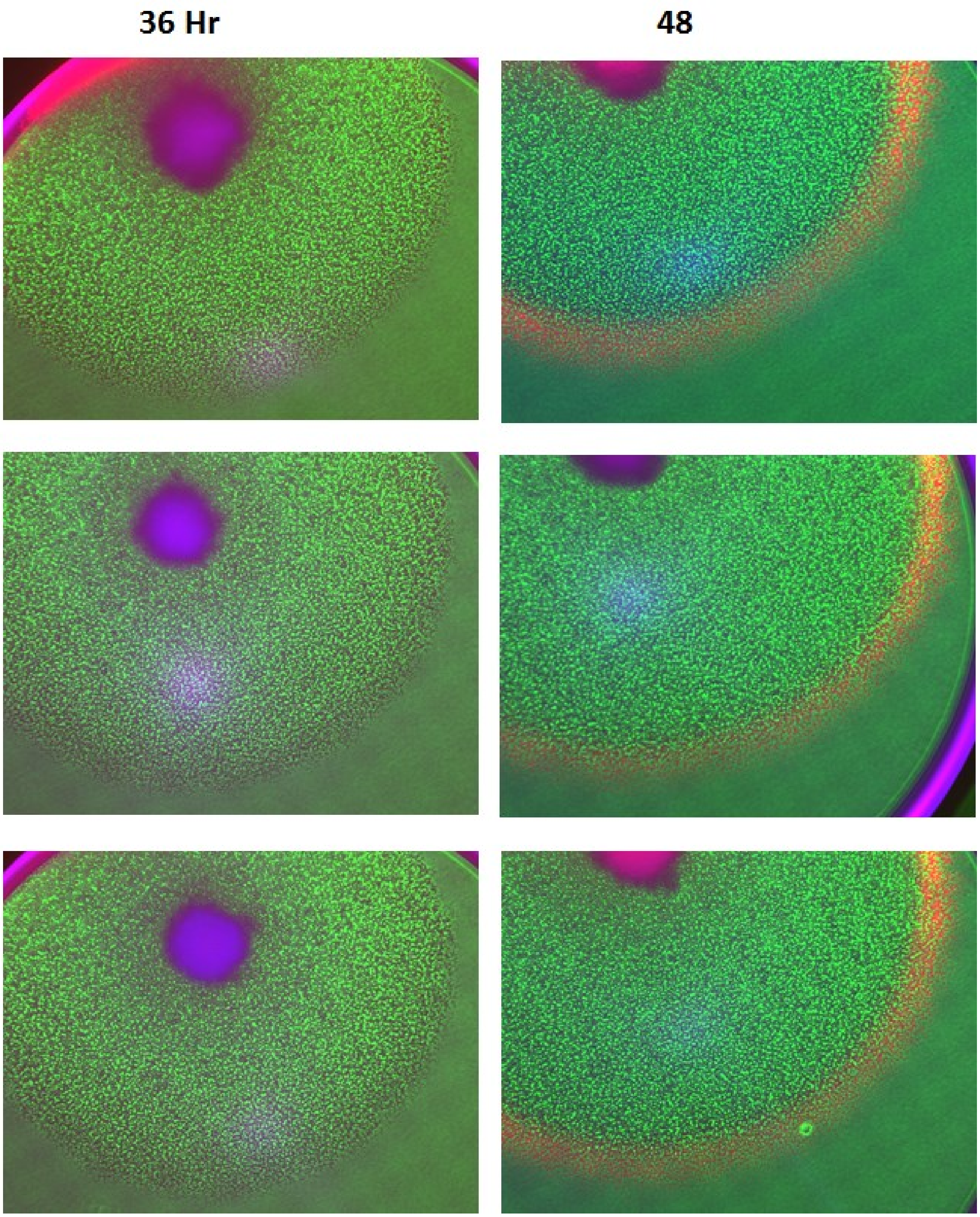

Figure S15: **Pattern formation for single colonies per droplet.**

MC4100Z1 colonies containing the synthetic gene circuit were prepared as in Main Fig. 1. These three colonies (a-c) were the sole colonies in their respective droplets. Raw (1.7 mm X 1.4 mm) composite images were obtained at 36 (left) and 48 (right) hrs after incubation at 30°C. The color scheme is as described in Movie S1. As is evident, the observed ring patterns are larger and appear later (48 hrs versus 36 hrs) than typical colonies in Main Fig. 3, which were not the sole colonies in their respective droplets.

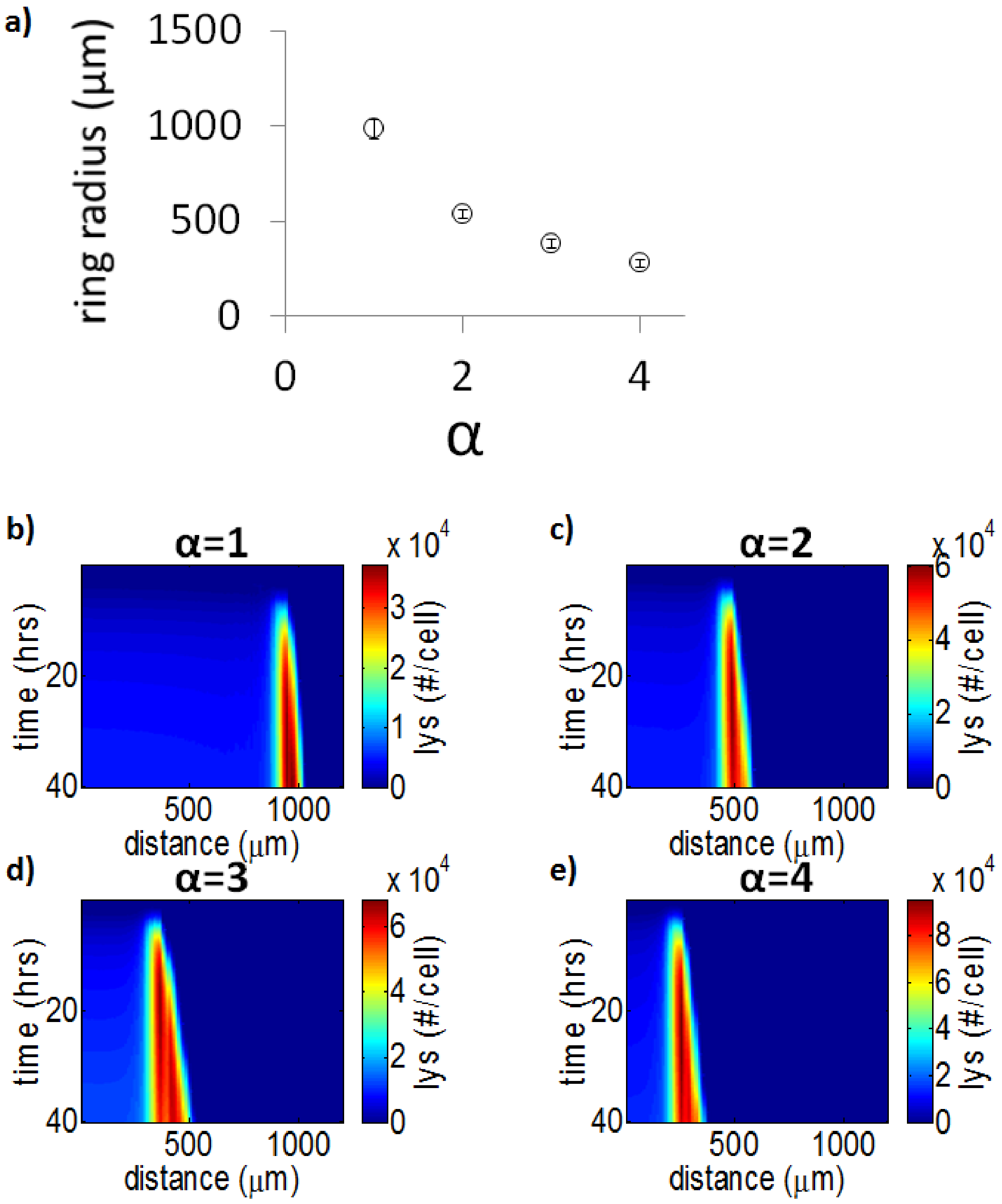

Figure S16: **Effect of perturbing gene expression capacity** φ**.** φ was perturbed by multiplicative factor α (base condition: α=1).

a) Average mCherry ring radii obtained at 25 hours for 1D simulations of microcolonies growing for α=1, 2, 3, and 4. All of the mCherry ring radii were calculated in the same manner as described in Main Fig. 1. The error bars represent standard error among eight replicates.

b)-e) Heat maps displaying T7 lysozyme for varying distance (x-axis) over time (y-axis) for the simulations with α=1 (b), 2 (c), 3 (d), and 4 (e). The intensity values represent the number of T7 lysozyme molecules per cell across the 1D spatial domain.

IV. Supplementary Tables

Table S1. Definition of model parameters and the base parameters

Parameter	Description	Basal Value
k_{T0}	Synthesis rate of T7 RNAP	20 #/(cell·min)
K_T	Half-activation threshold of T7 RNAP	10 #/cell (Tan et al, 2009)
d_T	Decay rate of T7 RNAP	5*10^{-3} /min (Tan et al, 2009)†
k_{A0}	Synthesis rate of AHL	32 #/(cell·min)
K_A	Half-activation threshold of AHL	20 nM (Collins et al, 2006)††
d_A	Decay rate of AHL	5*10^{-3} /min (You et al, 2004)
k_{L0}	Synthesis rate of T7 lysozyme	95 #/(cell·min)
K_P	Half-inhibition threshold of T-L complex	50 #/cell
K_{TL}	Dissociation constant for T-L complex	0.02 # (Kumar & Patel, 1997)
d_L	Decay rate of T7 lysozyme	2.4*10^{-4} /min
D_a	Diffusivity of AHL	6.7*10^{5} μm^{2}/min
θ_g	Cell division probability parameter	767
θ_m	Cell movement probability parameter	2*10^{4}
m	Hill coefficient of AHL binding to the *pluxI* promoter	2
K_ϕ	Half-activation distance for synthesis	6.5Δ
ϕ_0	Basal synthesis	0.1
γ	Hill coefficient for distance dependency	5
h_L	Hill coefficient for T7 lysozyme burden	10

h_T	Hill coefficient for T7 RNAP burden	2
K_{Lg}	Half-inhibition level of T7 lysozyme on growth	$5*10^4$
K_{Tg}	Half-inhibition level of T7 RNAP on growth	$1.4*10^4$
K_{Lm}	Half-inhibition level of T7 lysozyme on motility	620
K_{Tm}	Half-inhibition level of T7 RNAP on motility	3
α	Multiplicate factor of ϕ	1

†- The value used in this publication was 0.003 1/min. Our value is within 2-fold.

††- The value measured in this publication was 10 nM. Our value is within 2-fold.

Table S2. Primers used in this study

Amplified Region	Direction	Sequence (5’→ 3’)
Reverse pT7-pLux-mCherry	Forward	ATTGAGAATTCTATAGTGAGTCGTATTATCATGA GTCACACTATTGTATCGCTGGGAAT
Reverse pT7-pLux-mCherry	Reverse	ACATAGCCAGTAACTCGAGGCTAGCTAGTCAAGCTTTT ACTTGTACAGCTCGTCC
LacI	Forward	ACTAGCTAGCCTCGAGTTACTGGCTATGTGATAGCGCC CGGAAGAGAGTC
LacI	Reverse	ATTGCGACGTCCCTCGCCGAAAATGACCCAGA
T7 lysozyme	Forward	ACTAAGACGTCCGCCCAGTCCTGCTC
T7 lysozyme	Reverse	ACGGAGCTAGCGGAAAGGAGGAAAGAAATAATGGCTC
LuxR Knockout	Forward	CAGTGTCGACGGATCCCCGAATAAACGCAAGG
LuxR Knockout	Reverse	TGTTGTCGACCTATAGTGAGTCGTATTATCATGAGTC
LuxI Knockout	Forward	CATTGTCGACTAATGTTGTTAAGCTTCGCGGCC
LuxI Knockout	Reverse	CCATGTCGACCGTACTTAATTTTTAAAGTATGGGCAA

V. References

Collins CH, Leadbetter JR, Arnold FH (2006) Dual selection enhances the signaling specificity of a variant of the quorum-sensing transcriptional activator LuxR. *Nature biotechnology* **24:** 708-712

Kumar A, Patel SS (1997) Inhibition of T7 RNA polymerase: transcription initiation and transition from initiation to elongation are inhibited by T7 lysozyme via a ternary complex with RNA polymerase and promoter DNA. *Biochemistry* **36:** 13954-13962

Studier FW (1991) Use of bacteriophage T7 lysozyme to improve an inducible T7 expression system. *J Mol Biol* **219:** 37-44

Tan C, Marguet P, You L (2009) Emergent bistability by a growth-modulating positive feedback circuit. *Nat Chem Biol* **5:** 842-848

You L, Cox RS, 3rd, Weiss R, Arnold FH (2004) Programmed population control by cell-cell communication and regulated killing. *Nature* **428:** 868-871
