# Supplementary figures and images for: Temporal control of self-organized pattern formation without morphogen gradients in bacteria
Source: Mol Syst Biol. 2013 Oct 8;9:697. doi: 10.1038/msb.2013.55 (PMC3817405; doi:10.1038/msb.2013.55)

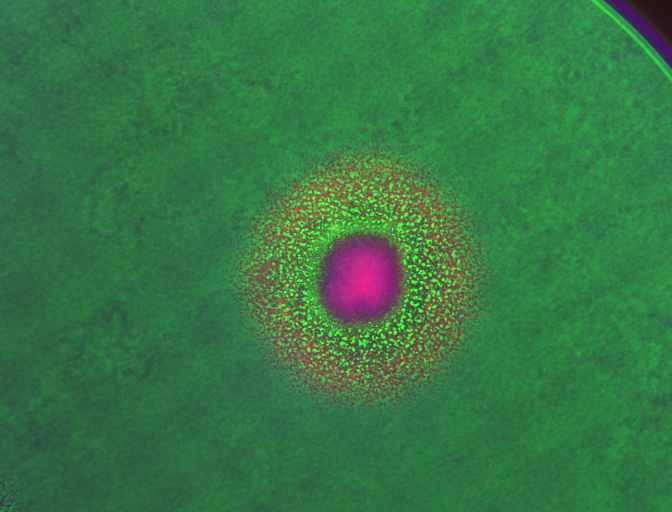

Supplement: Source Code and Raw Data for Main Figures [file msb201355-s4.zip › Paynemanuscript_Code&Data_Final/Code/Fig1/ERawImage.tif]

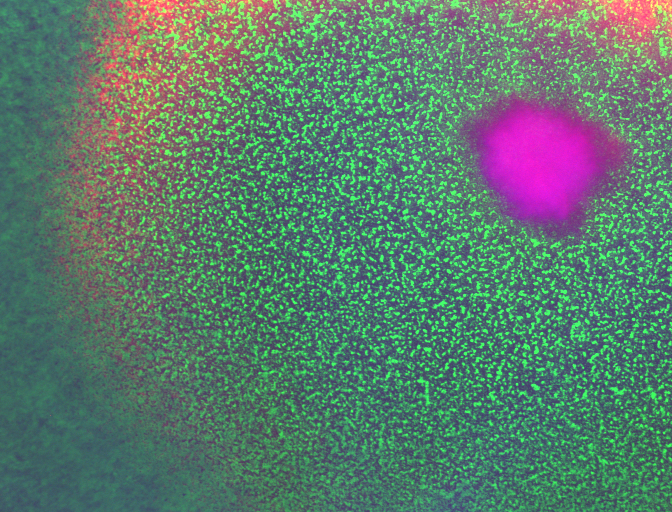

Supplement: Source Code and Raw Data for Main Figures [file msb201355-s4.zip › Paynemanuscript_Code&Data_Final/Code/Fig3/C&E/Replicate2.tif]

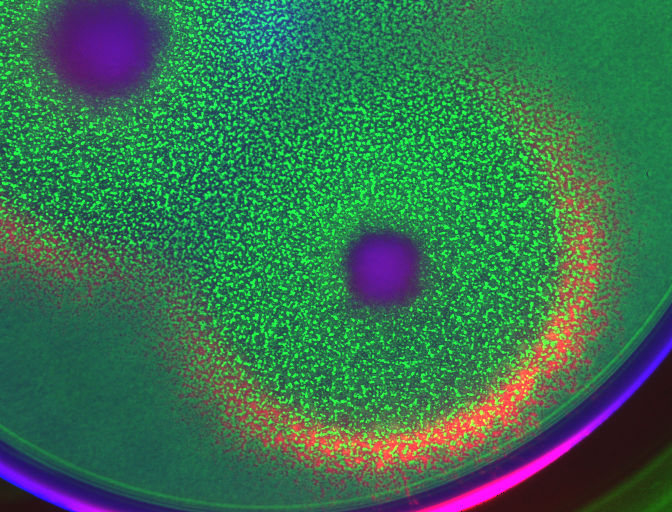

Supplement: Source Code and Raw Data for Main Figures [file msb201355-s4.zip › Paynemanuscript_Code&Data_Final/Code/Fig3/C&E/Replicate5.tif]

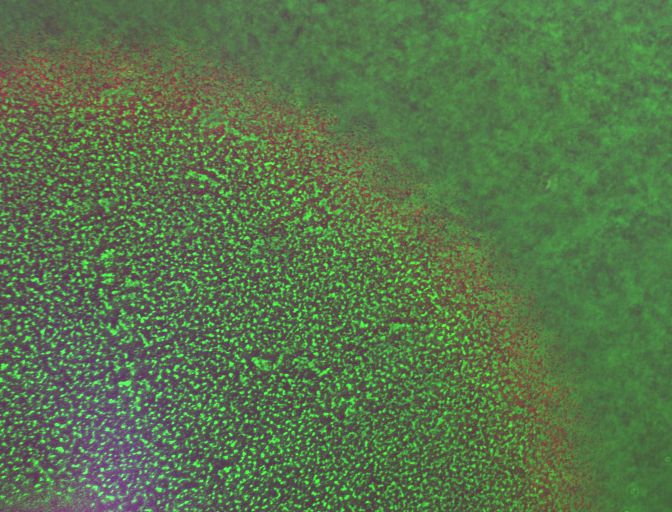

Supplement: Source Code and Raw Data for Main Figures [file msb201355-s4.zip › Paynemanuscript_Code&Data_Final/Code/Fig3/E/Replicate4.tif]

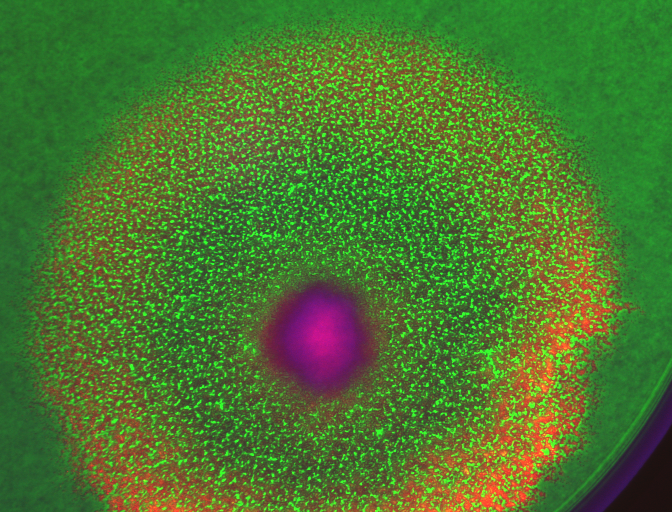

Supplement: Source Code and Raw Data for Main Figures [file msb201355-s4.zip › Paynemanuscript_Code&Data_Final/Code/Fig4/C&D/36HrImage.tif]

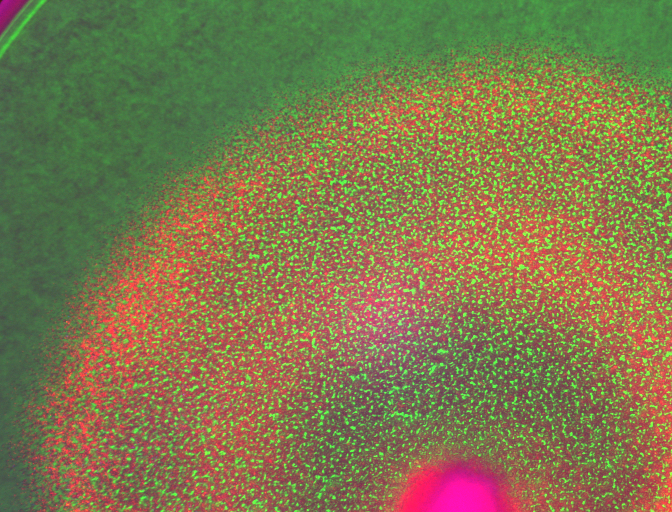

Supplement: Source Code and Raw Data for Main Figures [file msb201355-s4.zip › Paynemanuscript_Code&Data_Final/Code/Fig4/C&D/48HrImage.tif]

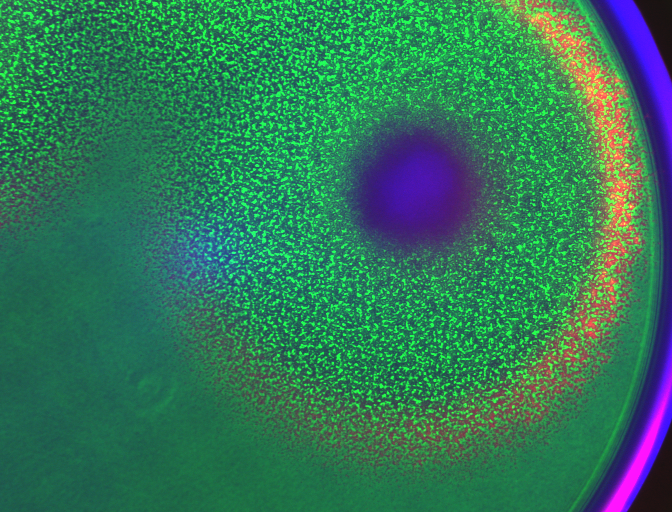

Supplement: Source Code and Raw Data for Main Figures [file msb201355-s4.zip › Paynemanuscript_Code&Data_Final/Data/Fig3/C/0nM_AHL/Replicate1.tif]

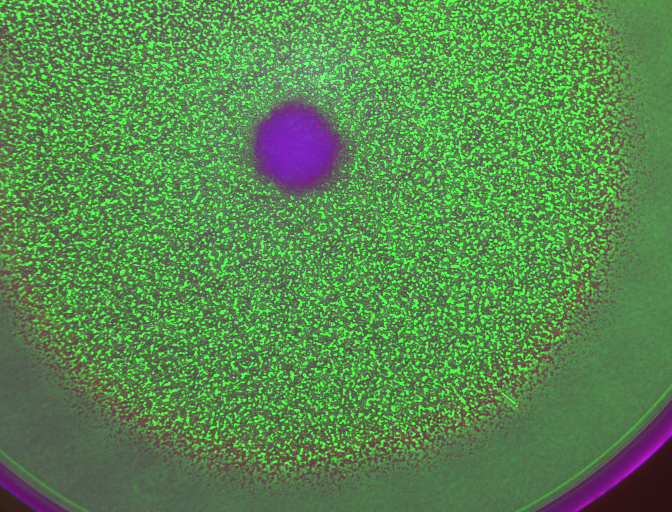

Supplement: Source Code and Raw Data for Main Figures [file msb201355-s4.zip › Paynemanuscript_Code&Data_Final/Data/Fig3/C/0nM_AHL/Replicate2.tif]

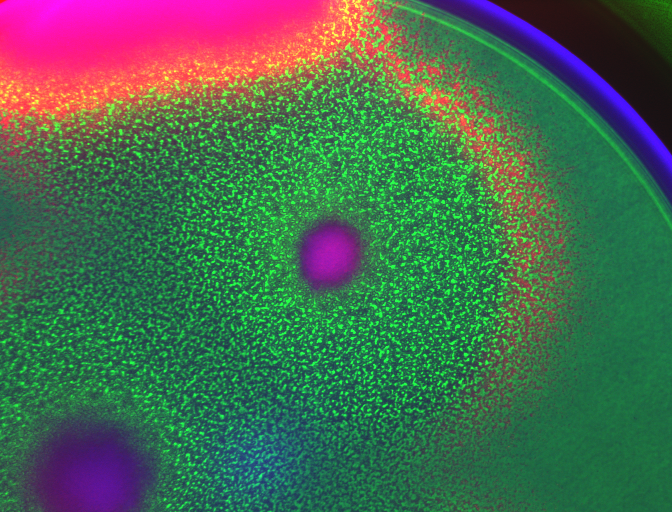

Supplement: Source Code and Raw Data for Main Figures [file msb201355-s4.zip › Paynemanuscript_Code&Data_Final/Data/Fig3/C/0nM_AHL/Replicate3.tif]

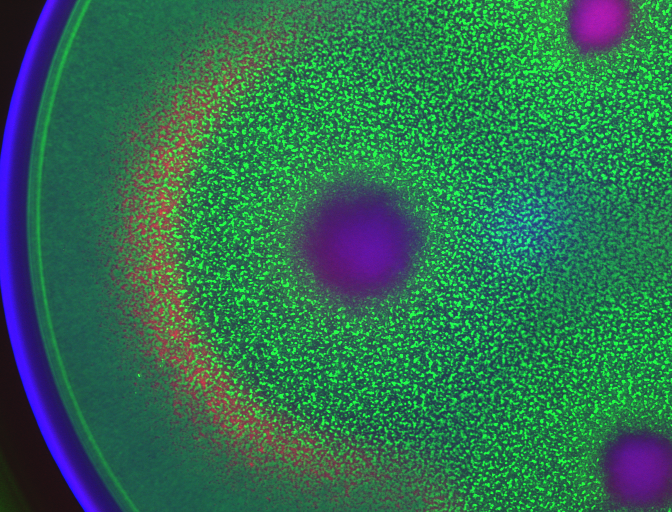

Supplement: Source Code and Raw Data for Main Figures [file msb201355-s4.zip › Paynemanuscript_Code&Data_Final/Data/Fig3/C/0nM_AHL/Replicate4.tif]

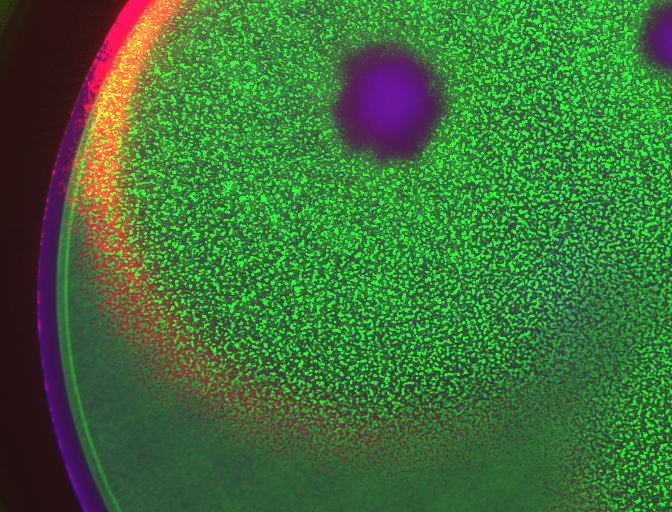

Supplement: Source Code and Raw Data for Main Figures [file msb201355-s4.zip › Paynemanuscript_Code&Data_Final/Data/Fig3/C/0nM_AHL/Replicate6.tif]

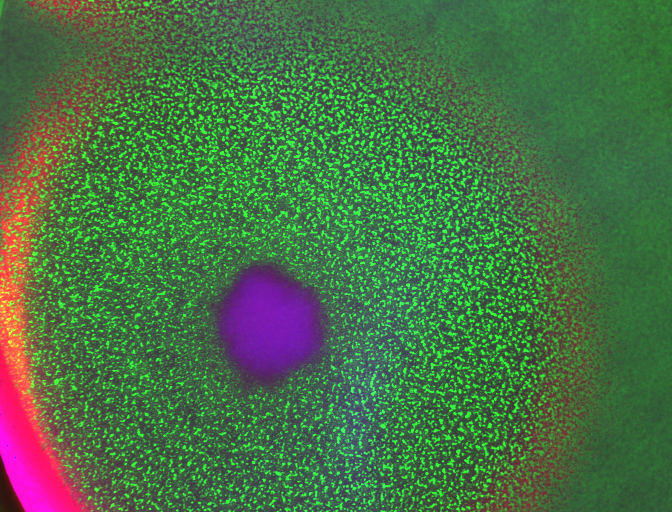

Supplement: Source Code and Raw Data for Main Figures [file msb201355-s4.zip › Paynemanuscript_Code&Data_Final/Data/Fig3/C/0nM_AHL/Replicate7.tif]

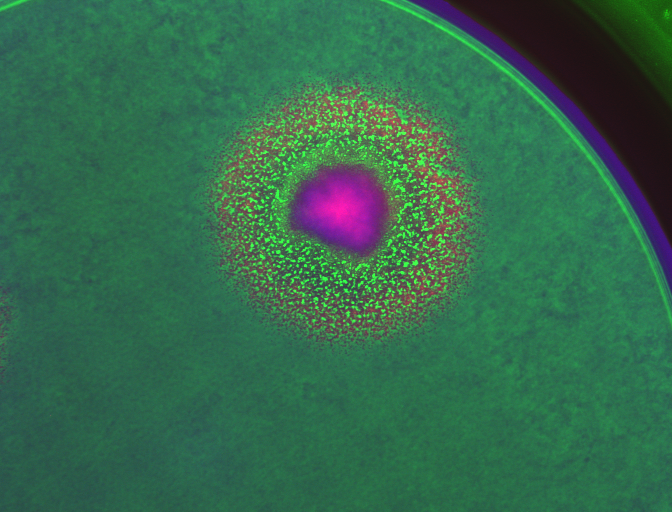

Supplement: Source Code and Raw Data for Main Figures [file msb201355-s4.zip › Paynemanuscript_Code&Data_Final/Data/Fig3/C/100nM_AHL/Replicate2.tif]

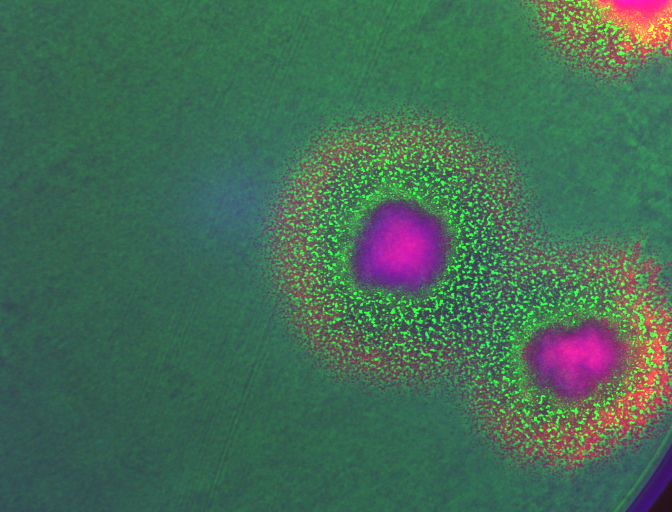

Supplement: Source Code and Raw Data for Main Figures [file msb201355-s4.zip › Paynemanuscript_Code&Data_Final/Data/Fig3/C/100nM_AHL/Replicate3.tif]

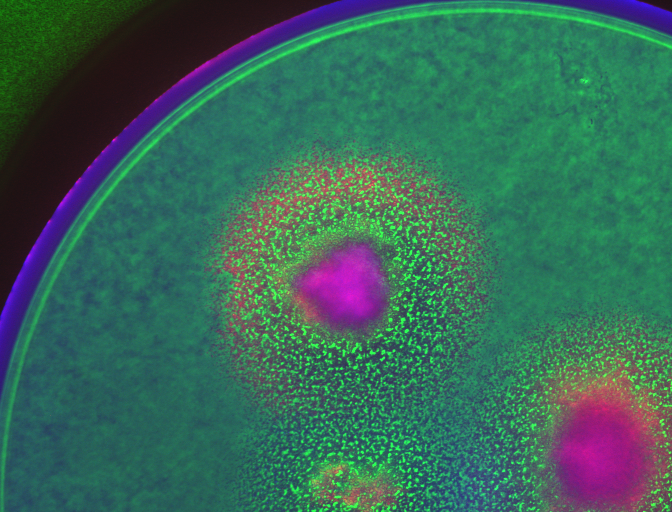

Supplement: Source Code and Raw Data for Main Figures [file msb201355-s4.zip › Paynemanuscript_Code&Data_Final/Data/Fig3/C/100nM_AHL/Replicate4.tif]

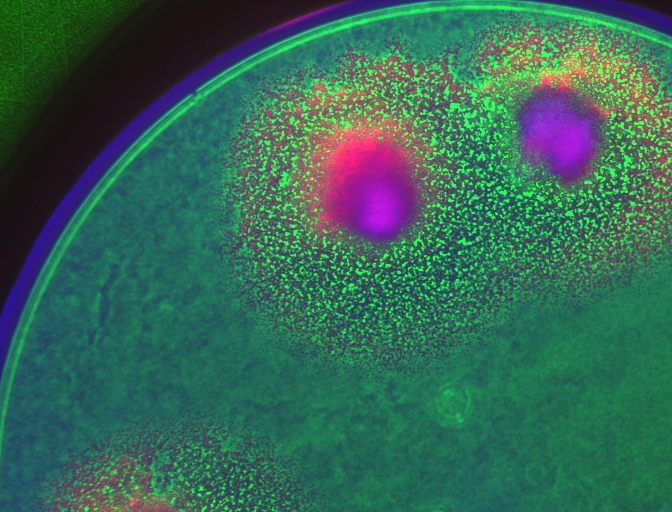

Supplement: Source Code and Raw Data for Main Figures [file msb201355-s4.zip › Paynemanuscript_Code&Data_Final/Data/Fig3/C/100nM_AHL/Replicate5.tif]

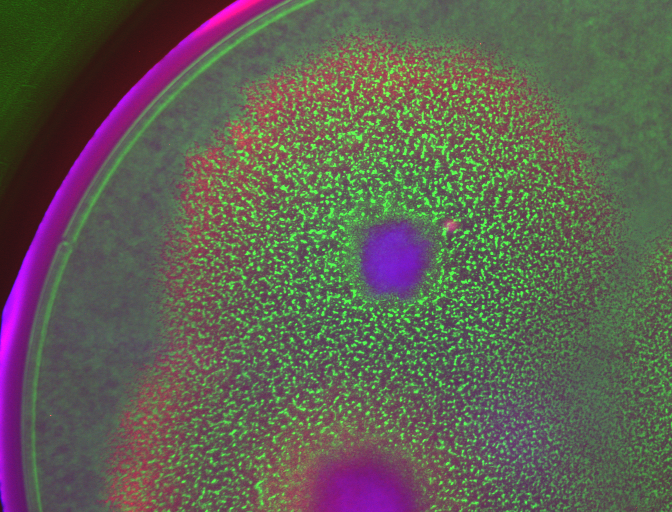

Supplement: Source Code and Raw Data for Main Figures [file msb201355-s4.zip › Paynemanuscript_Code&Data_Final/Data/Fig3/C/10nM_AHL/Replicate1.tif]

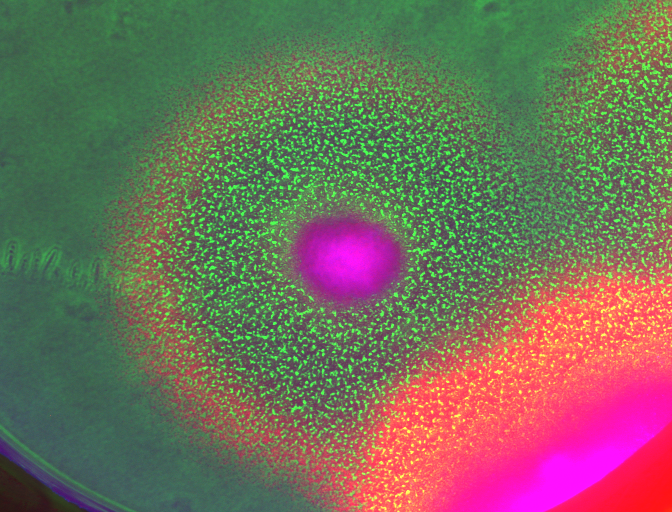

Supplement: Source Code and Raw Data for Main Figures [file msb201355-s4.zip › Paynemanuscript_Code&Data_Final/Data/Fig3/C/10nM_AHL/Replicate10.tif]

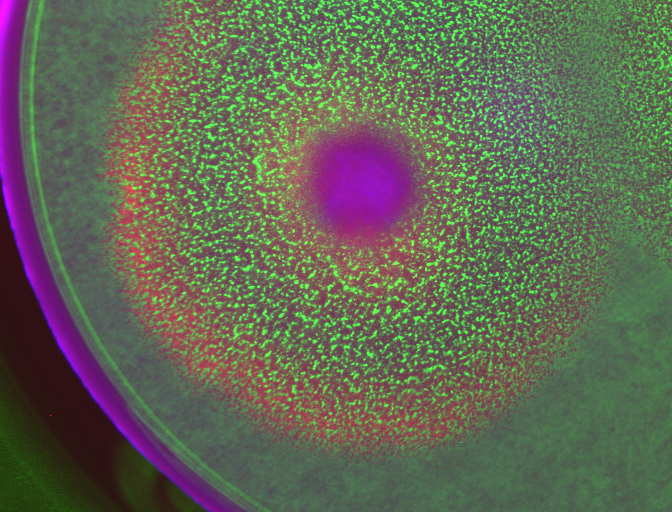

Supplement: Source Code and Raw Data for Main Figures [file msb201355-s4.zip › Paynemanuscript_Code&Data_Final/Data/Fig3/C/10nM_AHL/Replicate2.tif]

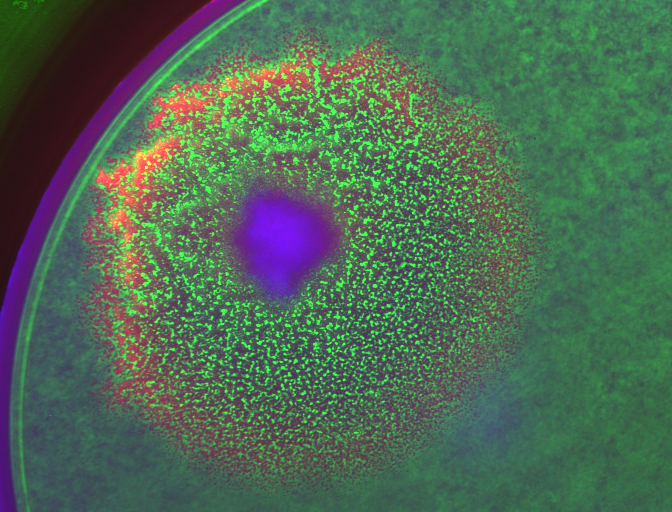

Supplement: Source Code and Raw Data for Main Figures [file msb201355-s4.zip › Paynemanuscript_Code&Data_Final/Data/Fig3/C/10nM_AHL/Replicate3.tif]

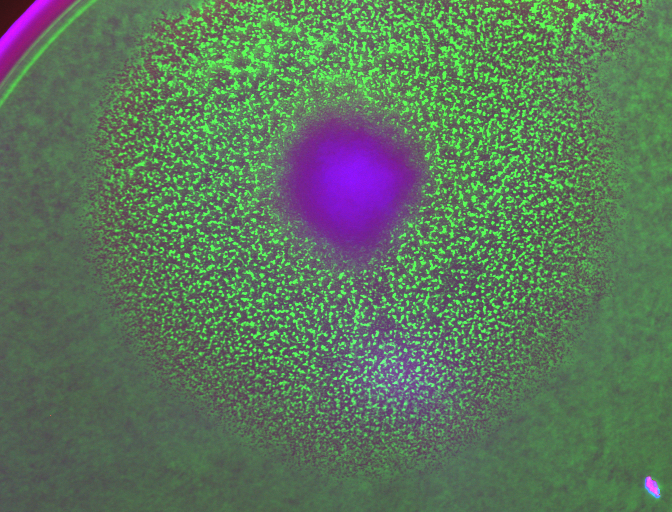

Supplement: Source Code and Raw Data for Main Figures [file msb201355-s4.zip › Paynemanuscript_Code&Data_Final/Data/Fig3/C/10nM_AHL/Replicate4.tif]

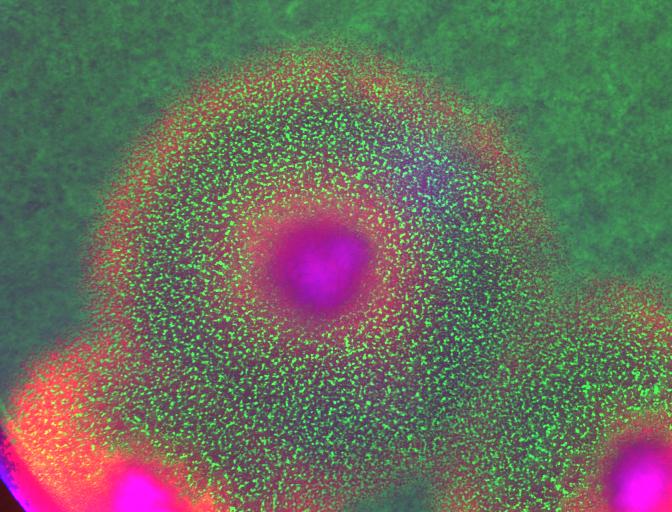

Supplement: Source Code and Raw Data for Main Figures [file msb201355-s4.zip › Paynemanuscript_Code&Data_Final/Data/Fig3/C/10nM_AHL/Replicate5.tif]

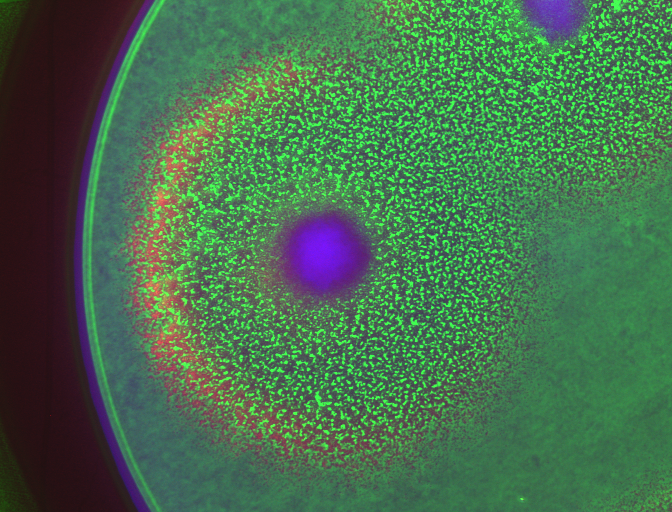

Supplement: Source Code and Raw Data for Main Figures [file msb201355-s4.zip › Paynemanuscript_Code&Data_Final/Data/Fig3/C/10nM_AHL/Replicate6.tif]

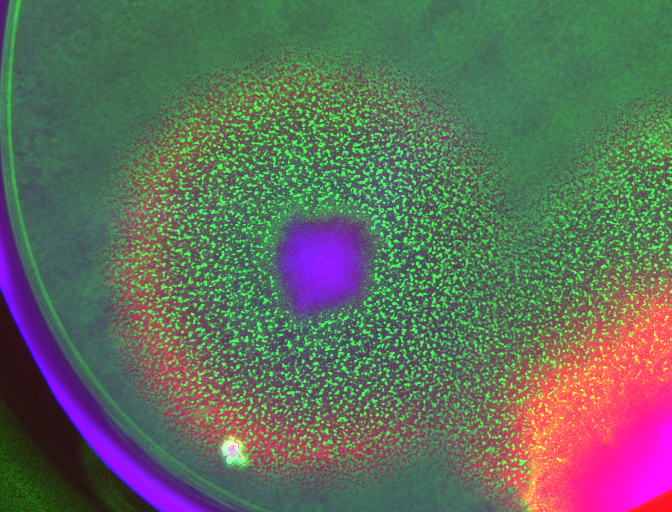

Supplement: Source Code and Raw Data for Main Figures [file msb201355-s4.zip › Paynemanuscript_Code&Data_Final/Data/Fig3/C/10nM_AHL/Replicate7.tif]

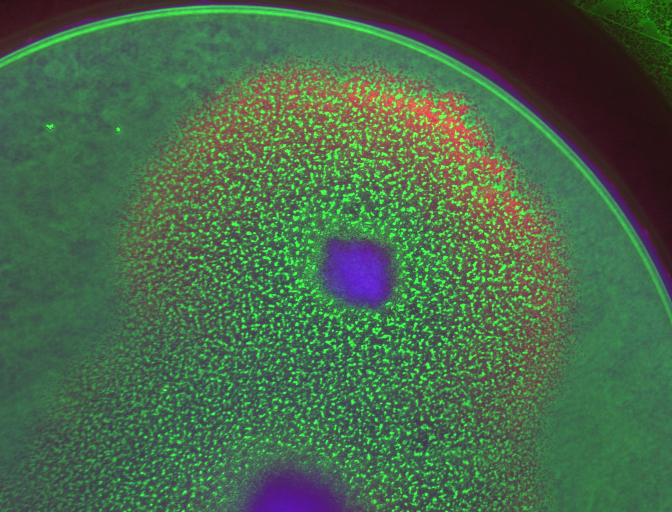

Supplement: Source Code and Raw Data for Main Figures [file msb201355-s4.zip › Paynemanuscript_Code&Data_Final/Data/Fig3/C/10nM_AHL/Replicate8.tif]

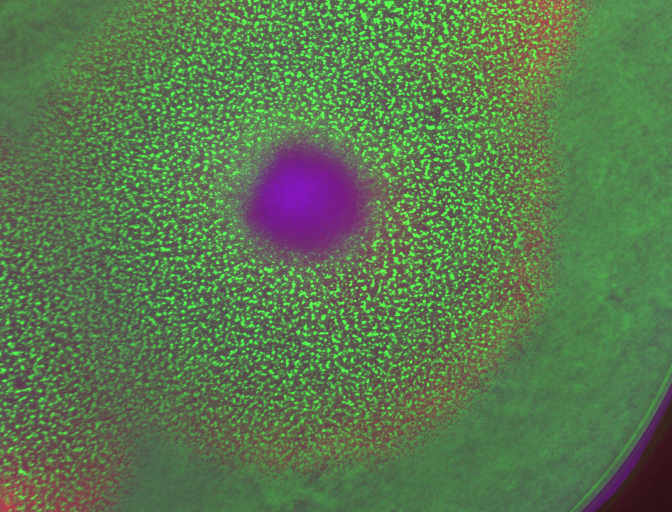

Supplement: Source Code and Raw Data for Main Figures [file msb201355-s4.zip › Paynemanuscript_Code&Data_Final/Data/Fig3/C/10nM_AHL/Replicate9.tif]

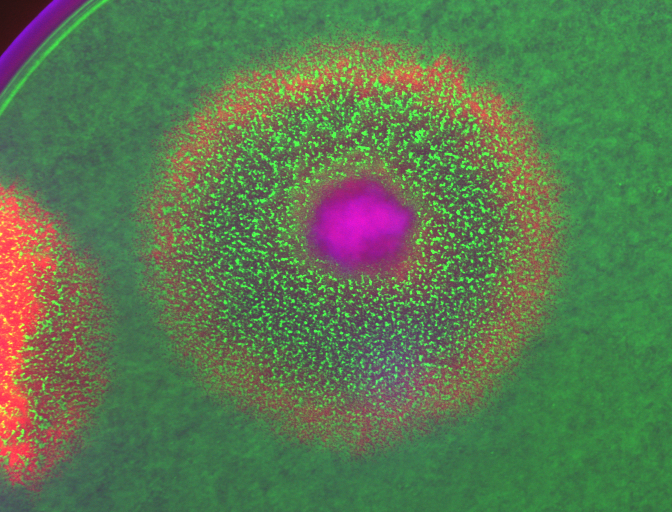

Supplement: Source Code and Raw Data for Main Figures [file msb201355-s4.zip › Paynemanuscript_Code&Data_Final/Data/Fig3/C/30nM_AHL/Replicate1.tif]

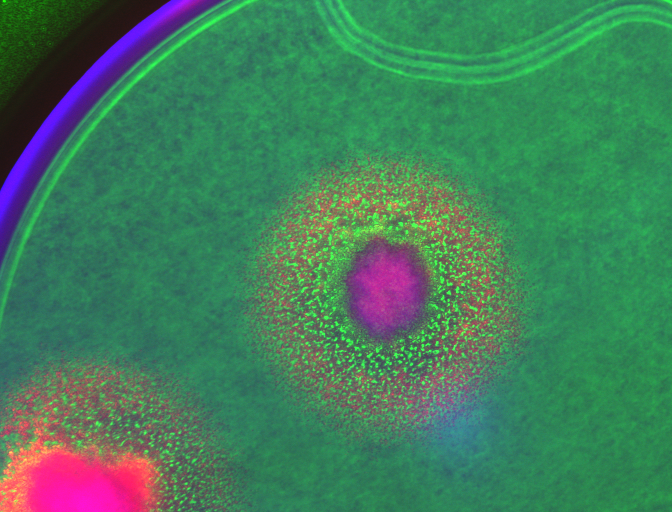

Supplement: Source Code and Raw Data for Main Figures [file msb201355-s4.zip › Paynemanuscript_Code&Data_Final/Data/Fig3/C/30nM_AHL/Replicate2.tif]

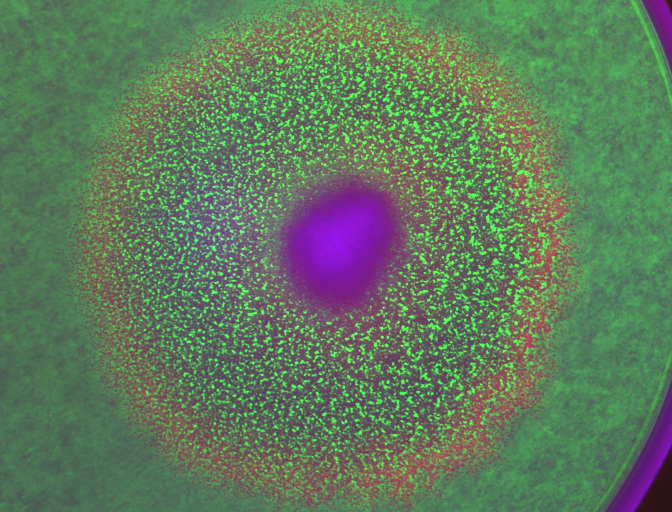

Supplement: Source Code and Raw Data for Main Figures [file msb201355-s4.zip › Paynemanuscript_Code&Data_Final/Data/Fig3/C/30nM_AHL/Replicate3.tif]

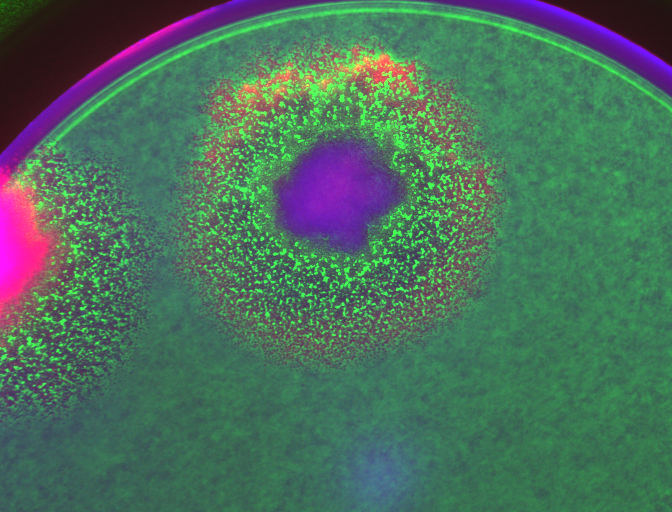

Supplement: Source Code and Raw Data for Main Figures [file msb201355-s4.zip › Paynemanuscript_Code&Data_Final/Data/Fig3/C/30nM_AHL/Replicate4.tif]

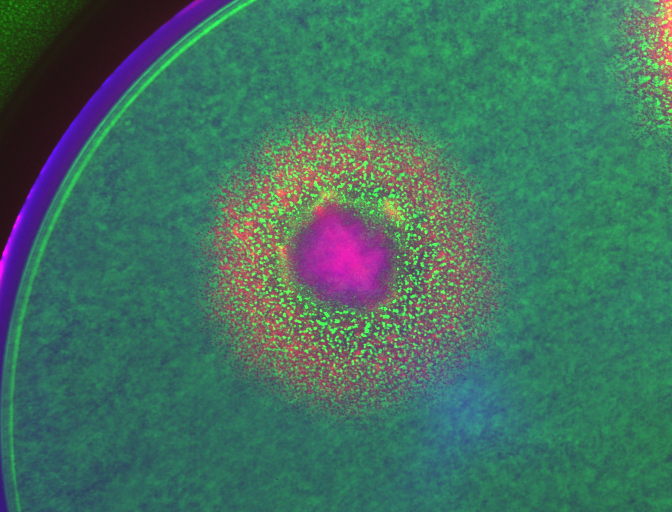

Supplement: Source Code and Raw Data for Main Figures [file msb201355-s4.zip › Paynemanuscript_Code&Data_Final/Data/Fig3/C/30nM_AHL/Replicate5.tif]

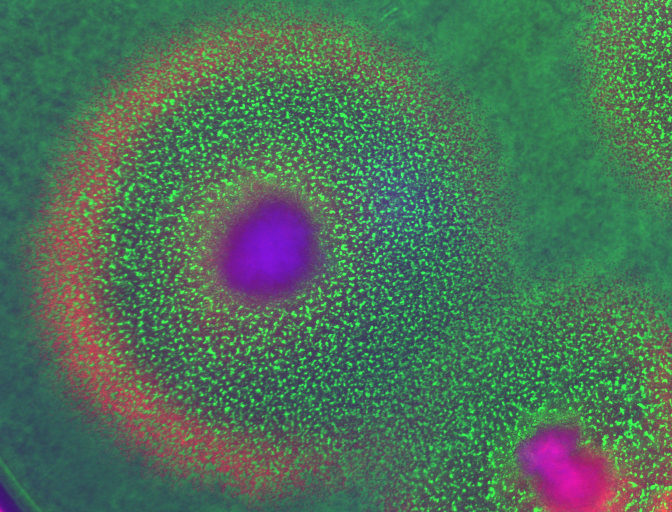

Supplement: Source Code and Raw Data for Main Figures [file msb201355-s4.zip › Paynemanuscript_Code&Data_Final/Data/Fig3/C/30nM_AHL/Replicate6.tif]

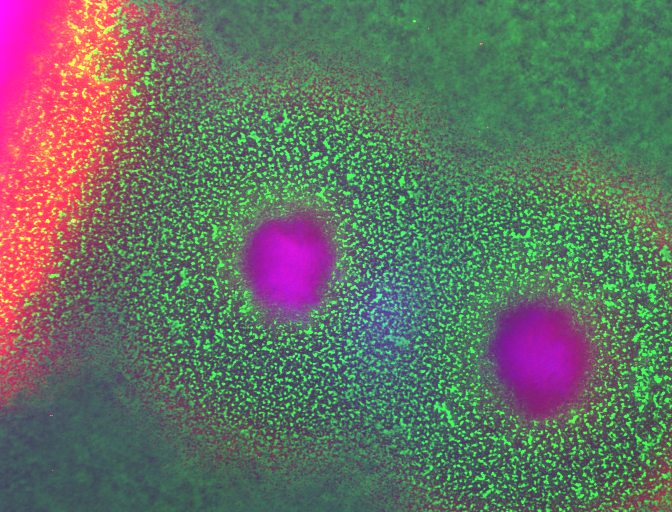

Supplement: Source Code and Raw Data for Main Figures [file msb201355-s4.zip › Paynemanuscript_Code&Data_Final/Data/Fig3/C/30nM_AHL/Replicate7.tif]

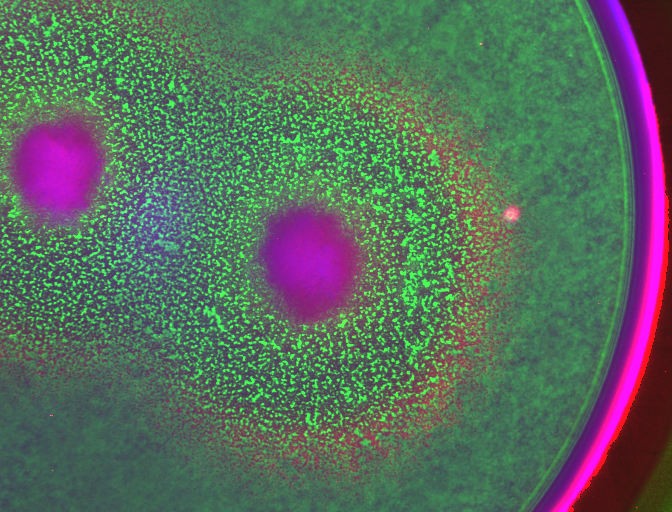

Supplement: Source Code and Raw Data for Main Figures [file msb201355-s4.zip › Paynemanuscript_Code&Data_Final/Data/Fig3/C/30nM_AHL/Replicate8.tif]

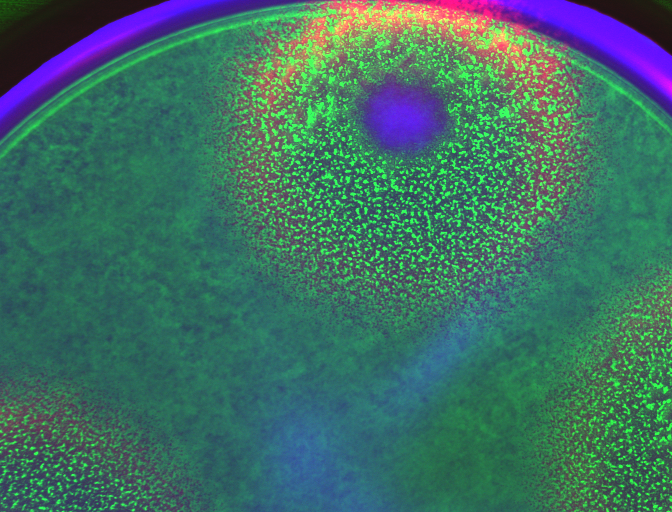

Supplement: Source Code and Raw Data for Main Figures [file msb201355-s4.zip › Paynemanuscript_Code&Data_Final/Data/Fig3/C/30nM_AHL/Replicate9.tif]

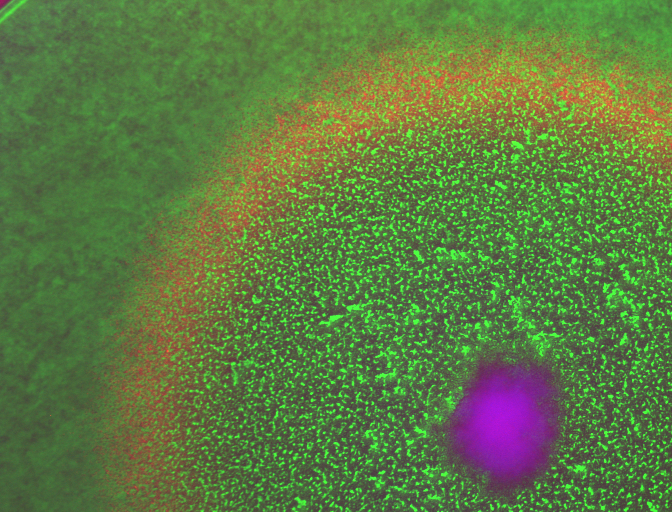

Supplement: Source Code and Raw Data for Main Figures [file msb201355-s4.zip › Paynemanuscript_Code&Data_Final/Data/Fig3/E/10ul/Replicate1.tif]

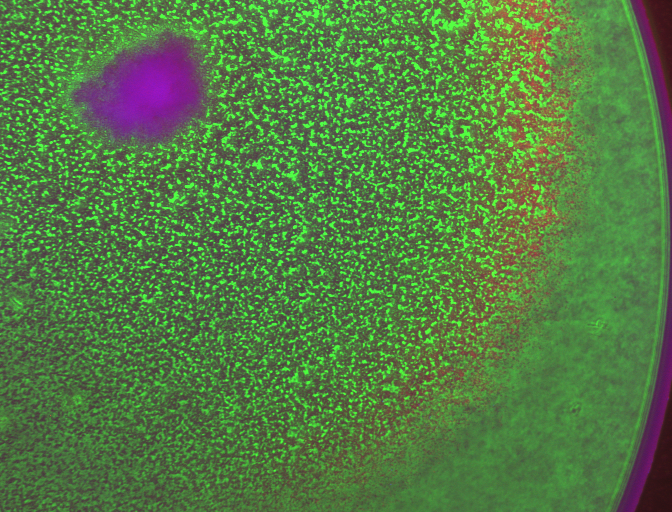

Supplement: Source Code and Raw Data for Main Figures [file msb201355-s4.zip › Paynemanuscript_Code&Data_Final/Data/Fig3/E/10ul/Replicate2.tif]

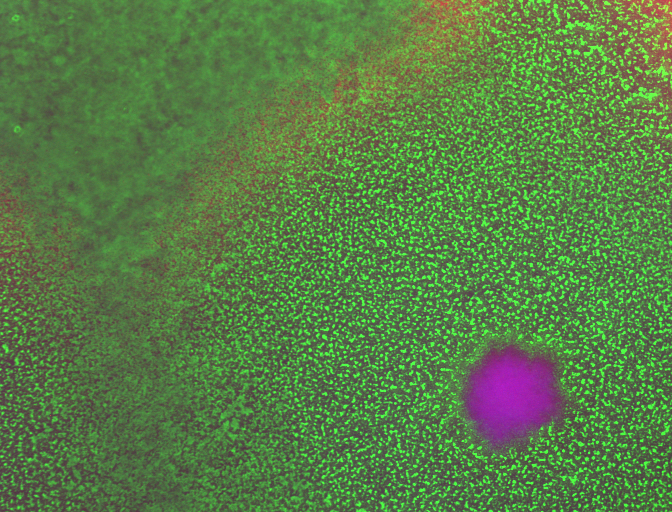

Supplement: Source Code and Raw Data for Main Figures [file msb201355-s4.zip › Paynemanuscript_Code&Data_Final/Data/Fig3/E/10ul/Replicate3.tif]

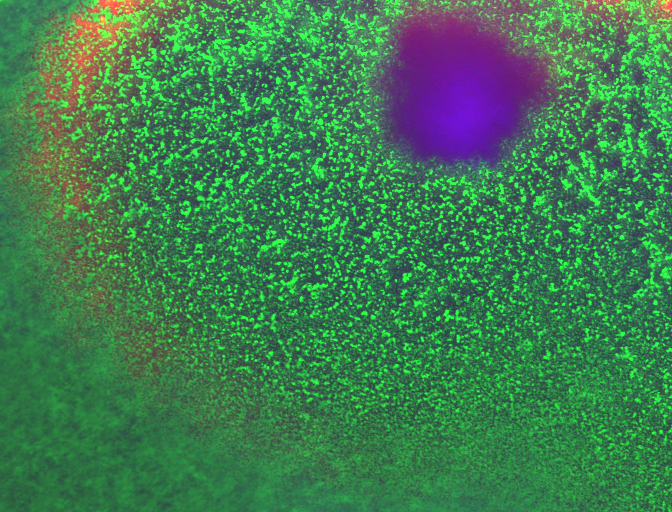

Supplement: Source Code and Raw Data for Main Figures [file msb201355-s4.zip › Paynemanuscript_Code&Data_Final/Data/Fig3/E/10ul/Replicate4.tif]

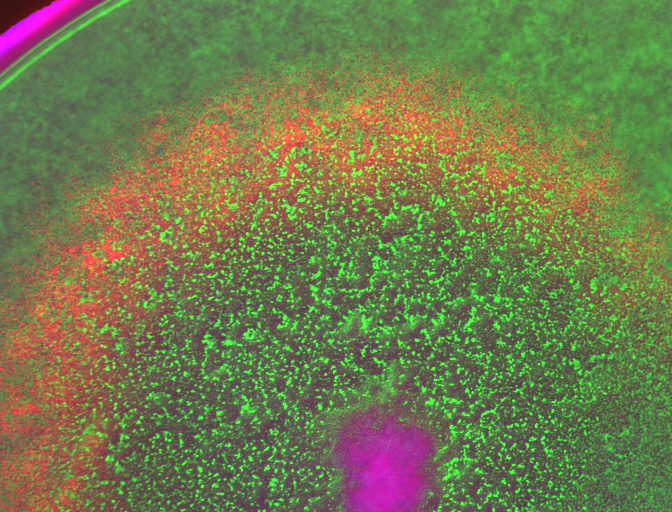

Supplement: Source Code and Raw Data for Main Figures [file msb201355-s4.zip › Paynemanuscript_Code&Data_Final/Data/Fig3/E/10ul/Replicate5.tif]

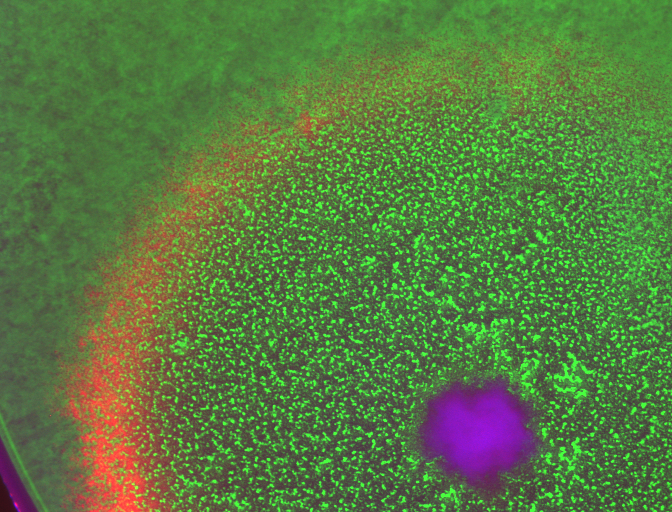

Supplement: Source Code and Raw Data for Main Figures [file msb201355-s4.zip › Paynemanuscript_Code&Data_Final/Data/Fig3/E/10ul/Replicate6.tif]

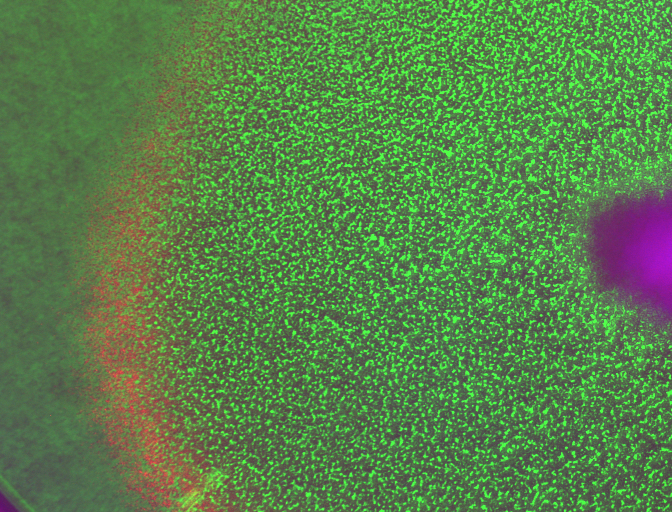

Supplement: Source Code and Raw Data for Main Figures [file msb201355-s4.zip › Paynemanuscript_Code&Data_Final/Data/Fig3/E/15ul/Replicate1.tif]

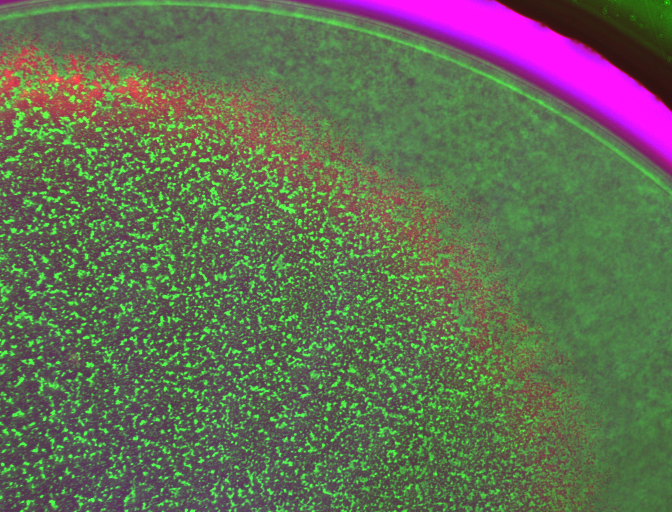

Supplement: Source Code and Raw Data for Main Figures [file msb201355-s4.zip › Paynemanuscript_Code&Data_Final/Data/Fig3/E/15ul/Replicate3.tif]

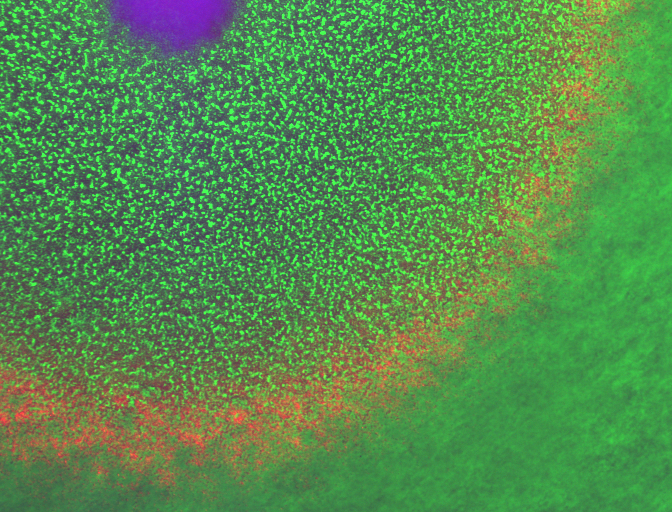

Supplement: Source Code and Raw Data for Main Figures [file msb201355-s4.zip › Paynemanuscript_Code&Data_Final/Data/Fig3/E/15ul/Replicate5.tif]

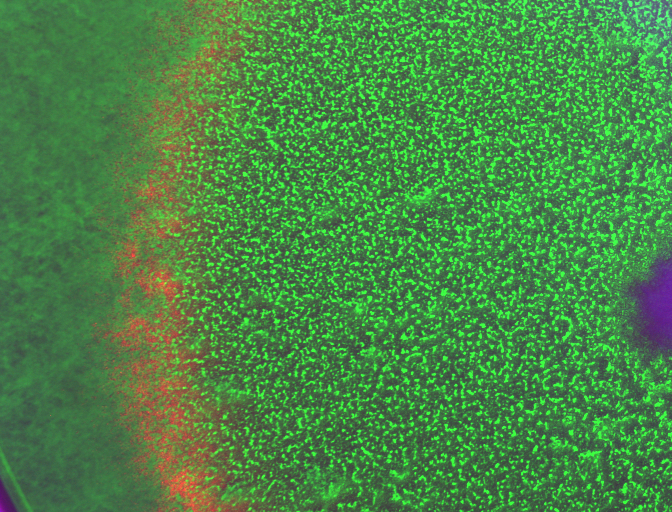

Supplement: Source Code and Raw Data for Main Figures [file msb201355-s4.zip › Paynemanuscript_Code&Data_Final/Data/Fig3/E/15ul/Replicate6.tif]

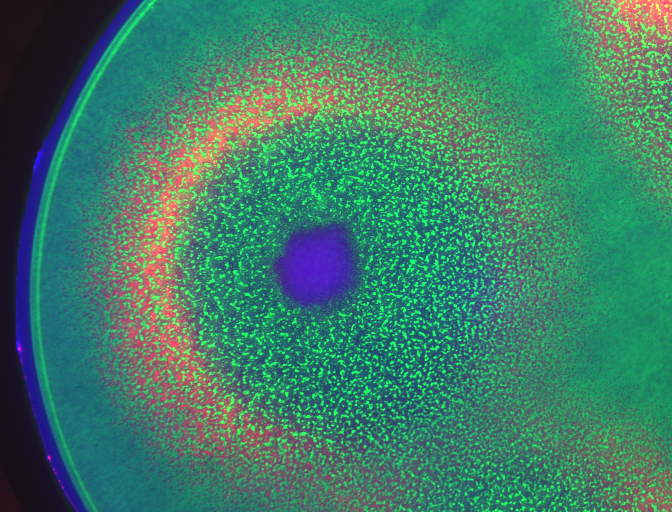

Supplement: Source Code and Raw Data for Main Figures [file msb201355-s4.zip › Paynemanuscript_Code&Data_Final/Data/Fig3/E/5ul/Replicate1.tif]

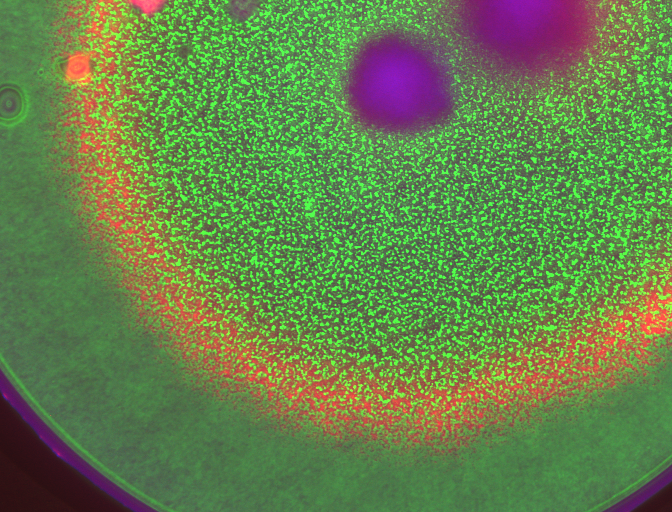

Supplement: Source Code and Raw Data for Main Figures [file msb201355-s4.zip › Paynemanuscript_Code&Data_Final/Data/Fig3/E/5ul/Replicate2.tif]

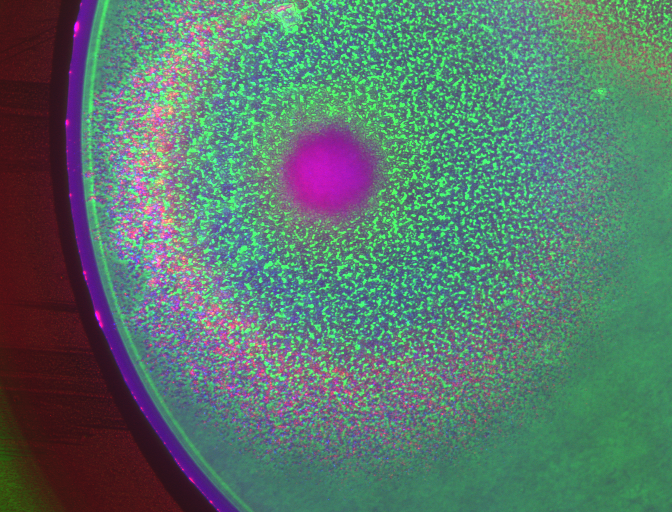

Supplement: Source Code and Raw Data for Main Figures [file msb201355-s4.zip › Paynemanuscript_Code&Data_Final/Data/Fig3/E/5ul/Replicate3.tif]
